# MSEA-Daily: A 4-km daily climate dataset for mainland Southeast Asia (1981–2014)

**DOI:** 10.1038/s41597-026-07653-x

**Published:** 2026-06-19

**Authors:** Teerachai Amnuaylojaroen, Atsamon Limsakul

**Affiliations:** 1 School of Energy and Environment, University of Phayao, Phayao, 56000 Thailand; 2 Atmospheric Pollution and Climate Change Research Units, School of Energy and Environment, University of Phayao, Phayao, 56000 Thailand; 3 Climate Change and Environmental Research Center, Pathum Thani, 12120 Thailand

## Abstract

Daily climate data at high spatial resolution is essential for impact assessment in mainland Southeast Asia, yet existing products either lack spatial detail or temporal coverage. We present MSEA-Daily, a gridded daily climate dataset at 4-km resolution spanning 1981–2014 for mainland Southeast Asia (Myanmar, Thailand, Laos, Cambodia, Vietnam). The dataset provides daily precipitation, maximum temperature, minimum temperature, and mean temperature that maintain strict consistency with TerraClimate monthly values while incorporating realistic daily variability from CHIRPS (precipitation) and AgERA5 (temperature). Independent validation against 77 GHCND stations across mainland Southeast Asia and peninsular Malaysia demonstrate moderate skill for precipitation and high-to-excellent skill for the three temperature variables, with minimum and mean temperature showing the strongest agreement against station observations. Residual systematic biases are dominated by a small cool bias in maximum temperature inherited from TerraClimate, near-zero bias in mean temperature, and spatially structured precipitation biases linked to orography. MSEA-Daily reproduces the seasonal cycle and the timing of monsoon onset and withdrawal across the dry, hot, rainy and transition seasons, although precipitation extremes at the 95th percentile and above are underestimated by approximately 10–15% relative to station observations. MSEA-Daily also reproduces the spatial pattern of the JJAS ENSO–monsoon teleconnection over mainland Southeast Asia, including the central Indochina dry/central Vietnamese wet precipitation dipole during El Niño years and the lagged temperature response over Indochina. The dataset enables integrated climate assessments requiring both monthly climatological consistency for trend analysis and daily resolution for process modeling, with primary applications in agricultural modeling, hydrological simulation, and climate extreme analysis.

## Background & Summary

High-resolution daily climate data is essential for agricultural planning, hydrological modeling, and climate impact assessments in mainland Southeast Asia, where over 230 million people depend on monsoon-driven agriculture and water resources^1^. Daily temporal resolution captures critical sub-monthly processes including monsoon onset timing, dry spell duration, and extreme precipitation events that determine crop yields, flood risk, and water availability but are invisible in monthly aggregates^2–4^. The region’s complex topography creates steep climate gradients where precipitation can vary by an order of magnitude over 10–20 km distances, requiring spatial resolutions of 5 km or finer to represent orographic effects and localized climate features^5–7^. However, mainland Southeast Asia has sparse station networks with fewer than 100 publicly available long-term daily climate stations across Thailand, Laos, Cambodia, Myanmar, and Vietnam combined^8^, creating fundamental barriers to data-based climate impact assessment and adaptation.

Several gridded daily climate datasets provide coverage over Southeast Asia, each with specific limitations that constrain applications. Climate Hazards Group InfraRed Precipitation with Station data (CHIRPS) offers daily precipitation at 0.05° resolution (~5.5 km) from 1981-present with demonstrated skill in the region^9^, but provides precipitation only without temperature variables essential for agricultural and hydrological models. ERA5-Land provides comprehensive meteorology at hourly resolution and 0.1° spacing (~9 km) from 1950-present^10^, but exhibits warm biases exceeding 2 °C in tropical monsoon regions^11^ and its coarser resolution misses topographic detail critical for steep terrain. Asian Precipitation - Highly Resolved Observational Data Integration Towards Evaluation (APHRODITE) provides daily precipitation and temperature at 0.25° resolution (~25 km) for 1951–2015^12^, but quality declines substantially after 2007 due to reduced station availability, the dataset ends in 2015, and the coarse resolution inadequately represents orographic precipitation gradients. Regional products like SA-OBS provide station-based daily data for parts of Southeast Asia^8^, but coverage remains limited to specific countries and time periods.

A specific gap exists for daily climate data that maintains consistency with established monthly climatologies used for long-term trend analysis. TerraClimate provides high-resolution (~4 km) monthly climate data from 1958-present using climate-aided interpolation of WorldClim climatologies with temporal anomalies from CRU Ts4.0 and JRA-55 reanalysis^13^. Many regional studies use TerraClimate monthly data for multi-decadal trend analysis and climatological characterization due to its spatial detail, long-term consistency, and integration of water balance variables. However, when these same studies require daily inputs for process-based models—common in agricultural water balance modeling, crop phenology simulation, and hydrological runoff generation—researchers must switch to different daily products, introducing inconsistencies between the monthly climatology used for trends and the daily data used for process modeling. This inconsistency complicates interpretation when monthly totals from daily products don’t match the monthly climatology being analyzed for trends.

We present MSEA-Daily, a gridded daily climate dataset at 4-km resolution spanning 1981–2014 for mainland Southeast Asia (4–24 °N, 92–110 °E). The dataset provides daily fields of precipitation, maximum temperature, minimum temperature, and mean temperature that maintain strict monthly conservation (precipitation) or mean-preservation (temperature) with TerraClimate monthly data, while incorporating realistic daily variability from observationally constrained reference datasets. For precipitation, we use CHIRPS daily patterns to distribute monthly TerraClimate totals, ensuring daily values sum exactly to TerraClimate monthly totals at each grid cell. For temperature, we add AgERA5 zero-mean daily anomalies to TerraClimate monthly means, guaranteeing daily values average to TerraClimate monthly means. This approach preserves TerraClimate’s spatial detail and climate-aided interpolation while enabling analysis of daily sequences, extreme events, and sub-monthly variability critical for impact assessments.

MSEA-Daily fills a specific niche for applications requiring both (1) consistency with TerraClimate monthly climatology for trend analysis and (2) daily temporal resolution for process modeling. Primary use cases include agricultural modeling where monthly precipitation trends inform long-term planning but daily sequences drive crop growth simulations^3,14^ hydrological modeling requiring daily inputs while maintaining water balance consistency with monthly climatologies^15,16^ climate extreme analysis needing daily values to calculate indices such as consecutive dry days and heat wave duration^17,18^ while preserving monthly context; and integrated assessments combining trend analysis (monthly scale) with impact modeling (daily scale). The dataset is not intended to replace CHIRPS, ERA5-Land, or other daily products for applications that don’t require TerraClimate consistency, nor is it suitable for applications requiring sub-daily temporal resolution or data beyond 2014.

## Methods

MSEA-Daily was generated by temporally disaggregating TerraClimate monthly climate data to daily resolution while preserving monthly totals (precipitation) or monthly means (temperature variables including mean, maximum, and minimum temperature). The disaggregation used daily temporal patterns from CHIRPS (precipitation) and AgERA5 (temperature), which were remapped to the TerraClimate grid and combined to produce daily fields that maintained strict consistency with TerraClimate monthly values. This two-tier approach distinguished precipitation (multiplicative scaling with intensity enhancement) from temperature (additive anomaly method) to accommodate their different statistical properties.

### Data sources

The Global Historical Climatology Network-Daily (GHCND) database^19,20^ was used as the independent station-based validation reference for MSEA-Daily; production of MSEA-Daily itself does not depend on GHCND. We extracted daily precipitation, maximum, and minimum temperature records for stations within 4–24 °N/92–110 °E covering 1981–2014, using the 19-flag NOAA quality assurance described by Durre *et al*.^20^ to screen the records. The 77 in-domain stations retained after screening are tabulated in Table 1. GHCND daily station files were downloaded from NOAA NCEI at https://www.ncei.noaa.gov/data/global-historical-climatology-network-daily/ (dataset https://doi.org/10.7289/V5D21VHZ).

**Table 1:** Distribution of the 77 GHCND validation stations used in the independent evaluation of MSEA-Daily, by country and elevation band.

Country	0–200 m	200–500 m	500–1000 m	>1000 m	Total	Station elevation range (m)
Thailand	40	7	0	0	47	2–416
Vietnam	12	2	1	0	15	2–676
Laos	5	2	0	0	7	52–326
Myanmar	4	0	0	0	4	8–76
Malaysia	3	0	0	0	3	3–8
Cambodia	1	0	0	0	1	12
Total	65	11	0	0	77	2–676

TerraClimate^13^ provided the monthly precipitation totals and monthly mean/maximum/minimum 2-m air-temperature fields used as the conservation anchor of MSEA-Daily, at 1/24° (~4 km) resolution for 1981–2014 across 4–24 °N/92–110 °E. TerraClimate’s climate-aided interpolation methodology, parent products (WorldClim 2.0, CRU Ts4.0, JRA-55) and global validation are described by Abatzoglou *et al*.^13^; the present study used the published TerraClimate fields without modification. TerraClimate monthly data were obtained from the Climatology Lab THREDDS server at https://www.climatologylab.org/terraclimate.html (dataset https://doi.org/10.1038/sdata.2017.191). TerraClimate’s adoption record and reliability for mainland Southeast Asia are discussed in Usage Notes — Validation Considerations.

Climate Hazards Group InfraRed Precipitation with Station data (CHIRPS) version 2.0^9^ provided the daily precipitation patterns used to disaggregate TerraClimate monthly totals. CHIRPS daily fields at 0.05° (~5.5 km) resolution covering 1981–2014 were used; production methodology, known limitations (extreme-event under-representation, complex-topography effects, coastal sea-breeze under-resolution) and regional validation are described by Funk *et al*.^9^, Dinku *et al*.^21^ and Cavalcante *et al*.^22^. CHIRPS v2.0 daily NetCDF files were downloaded from the Climate Hazards Center file server at https://data.chc.ucsb.edu/products/CHIRPS-2.0/global_daily/netcdf/p05/ (dataset https://doi.org/10.15780/G2RP4Q).

AgERA5^23^ provided the daily 2-m air-temperature anomalies used to add sub-monthly variability to TerraClimate monthly means. We retrieved the daily mean, maximum, and minimum 2-m air temperature for 1981–2014 from the Copernicus Climate Data Store using the cdsapi Python client against dataset identifier sis-agrometeorological-indicators (variables 2m_temperature with statistics 24_hour_mean, 24_hour_maximum, and 24_hour_minimum; https://cds.climate.copernicus.eu/datasets/sis-agrometeorological-indicators; dataset https://doi.org/10.24381/cds.6c68c9bb). Production methodology and known biases of AgERA5 are described by Boogaard *et al*.^23^. Because the temperature disaggregation adds zero-mean AgERA5 anomalies to TerraClimate monthly means (see Temperature Disaggregation below), any systematic bias in AgERA5 absolute temperatures is removed by construction and does not propagate into MSEA-Daily.

### Data preprocessing

All source datasets were harmonized to the TerraClimate grid (1/24° resolution) using bilinear interpolation^24^. For each TerraClimate grid point, we computed weighted averages from the four nearest source grid cells with weights determined by inverse distance^25,26^. Monthly aggregates were then computed from daily fields to establish reference values for disaggregation:

$${P}_{\mathrm{month}}^{\mathrm{ref}}(m,{\rm{x}})=\sum _{d\in m}{P}^{\mathrm{CHIRPS}}(d,{\rm{x}})$$

$${\bar{T}}_{\mathrm{month}}^{{\rm{ref}}}(m,{\rm{x}})=\frac{1}{{\rm{| }}m{\rm{| }}}\sum _{d\in m}{T}^{\mathrm{ERA}5\mathrm{L}}(d,{\rm{x}})$$

where m denotes month, ${\rm{| }}m{\rm{| }}$ is days in month, and ${\bf{x}}$ denotes grid location. Detailed interpolation procedures are provided in Supplementary Methods S1.

### Precipitation disaggregation

Precipitation disaggregation distributed TerraClimate monthly totals according to CHIRPS daily patterns while enhancing wet-day intensity to better represent monsoon precipitation characteristics^27,28^. For each month and grid cell, we computed sharpened daily fractions:

$${f}_{\mathrm{sharp}}(d,{\rm{x}})=\frac{{[{P}^{\mathrm{CHIRPS}}(d,{\rm{x}})]}^{\alpha }}{\sum _{t\in m}{[{P}^{\mathrm{CHIRPS}}(t,{\rm{x}})]}^{\alpha }}$$

where α = 1.35 is a power transformation exponent that increases fractional allocation to high-precipitation days^29^. The exponent was optimized by maximizing a composite objective balancing overall skill (Kling-Gupta Efficiency)^30,31^, intensity representation (Simple Daily Intensity Index, 95th percentile)^17^, and wet-day frequency preservation against GHCND validation stations (Supplementary Methods S2). Daily disaggregated precipitation was computed as:

$$\hat{P}(d,{\rm{x}})={P}_{\mathrm{TC}}(m,{\rm{x}})\cdot {f}_{\mathrm{sharp}}(d,{\rm{x}})$$

where ${P}_{\mathrm{TC}}(m,{\rm{x}})$ is TerraClimate monthly total. This guaranteed exact conservation^32^: ${\sum }_{d\in m}\hat{P}(d,{\rm{x}})={P}_{\mathrm{TC}}(m,{\rm{x}})$.

When CHIRPS monthly total ≤1.0 mm or contains <2 wet days (threshold 0.1 mm/day), we assigned uniform daily fractions before power transformation. When CHIRPS showed zero precipitation but TerraClimate has non-zero totals, we distributed precipitation uniformly across a climatologically appropriate number of wet days derived from CHIRPS 1981–2010 monthly climatology^33^. Complete algorithms are in Supplementary Methods S2.

### Temperature disaggregation

Temperature disaggregation added AgERA5 daily anomalies to TerraClimate monthly means, preserving monthly averages while incorporating realistic daily variability^34,35^. For each month, we computed zero-mean daily anomalies:

$${\delta }^{(\cdot )}(d,{\rm{x}})={T}^{\mathrm{ERA}5\mathrm{L},(\cdot )}(d,{\rm{x}})-{\bar{T}}_{\mathrm{month}}^{\mathrm{ERA}5\mathrm{L},(\cdot )}(m,{\rm{x}})$$

where superscript (·) denotes temperature type (max, min, or mean). Daily disaggregated temperature was computed as:

$${\hat{T}}^{(\cdot )}(d,{\rm{x}})={T}_{\mathrm{TC}}^{(\cdot )}(m,{\rm{x}})+{\delta }^{(\cdot )}(d,{\rm{x}})$$

where ${T}_{\mathrm{TC}}^{(\cdot )}(m,{\rm{x}})$ is TerraClimate monthly mean. This preserved monthly means exactly^36,37^ $\frac{1}{{\rm{| }}m{\rm{| }}}{\sum }_{d\in m}{\hat{T}}^{(\cdot )}(d,{\rm{x}})={T}_{\mathrm{TC}}^{(\cdot )}(m,{\rm{x}})$. The method was applied identically for maximum, minimum, and mean temperature.

### Quality assurance

To conservation verification, we verified monthly conservation (precipitation) and mean-preservation (temperature variables) post-production for all 408 months ×~200,000 grid cells following standard quality control procedures for gridded climate datasets^38,39^. Target thresholds were |residual| < 0.1 mm/month for precipitation and <0.01 °C for temperature^34^. Across the full domain, median absolute errors were 0.003 mm/month (precipitation) and 0.001 °C (temperature), with 99.8% and 99.9% meeting thresholds respectively. Maximum errors occurred at coastal boundaries (<0.5 mm/month, <0.05 °C). For physical consistency checks, we verified (1) daily precipitation ≥0, (2) Tmax ≥ Tmin at all locations and times, (3) no physically implausible values (T < −20 °C or >50 °C, P > 500 mm/day, flagging <0.01% of values), and (4) no abrupt temporal discontinuities (>20 °C day-to-day temperature change or >200 mm/day precipitation change, with zero cases detected) following established quality assurance protocols^20,40^.

## Data Records

The MSEA-Daily dataset is publicly archived at Science Data Bank^41^ and consists of four primary climate variables provided as daily gridded fields in NetCDF format. The dataset covers mainland Southeast Asia including Myanmar, Thailand, Laos, Cambodia, and Vietnam over the geographic extent of 4–24 °N and 92–110 °E at a horizontal resolution of 1/24° (approximately 4 km or 0.04°). The grid comprises 432 longitude by 480 latitude cells, yielding approximately 110,135 land grid cells after ocean masking, using WGS84 geographic coordinates in an unprojected latitude-longitude system. The temporal coverage spans from January 1, 1981 to December 31, 2014, providing 12,418 daily time steps across 408 months. Time is encoded as days since 1981-01-01 00:00:00 UTC following standard CF conventions. Each of the four climate variables is stored in separate annual NetCDF files, creating a total of 136 files (34 years ×4 variables). Precipitation (precip) is provided in units of mm/day. Maximum temperature (tmax), minimum temperature (tmin), and mean temperature (tmean) are all provided in degrees Celsius. Files are named following the pattern daily_terraclimate_{variable}_{year}.nc, where variable is one of precip, tmax, tmin, or tmean, and year ranges from 1981 to 2014. For example, precipitation data for 1985 is stored in daily_terraclimate_precip_1985.nc, while maximum temperature for 2010 is stored in daily_terraclimate_tmax_2010.nc.

## Technical Validation

Technical validation of MSEA-Daily employs a two-tier approach that separates independent skill assessment from internal quality control. Independent validation compares MSEA-Daily against Global Historical Climatology Network-Daily (GHCND) station observations not used in dataset production^19^ and provides the primary assessment of dataset accuracy and transferability. To clarify whether MSEA-Daily mitigates, inherits, or amplifies the systematic biases of its source datasets, the two source fields used in disaggregation — CHIRPS for precipitation and AgERA5 for temperature — are additionally evaluated against the same 77-station GHCND population using identical across-station methodology and stratification (Source-Data Benchmarking). Internal consistency checks then compare MSEA-Daily against the CHIRPS and AgERA5 source datasets themselves; because CHIRPS supplies precipitation patterns and AgERA5 supplies temperature anomalies during disaggregation, high agreement is expected by construction, and these comparisons serve as quality control rather than independent validation (Internal Consistency Verification). Spatial and temporal validation patterns are presented in the Spatial Validation Patterns and Temporal Dynamics subsections, and the preservation of interannual variability is verified in the Preservation of Interannual Variability subsection.

### Validation framework and metrics

**Fig. 1:**
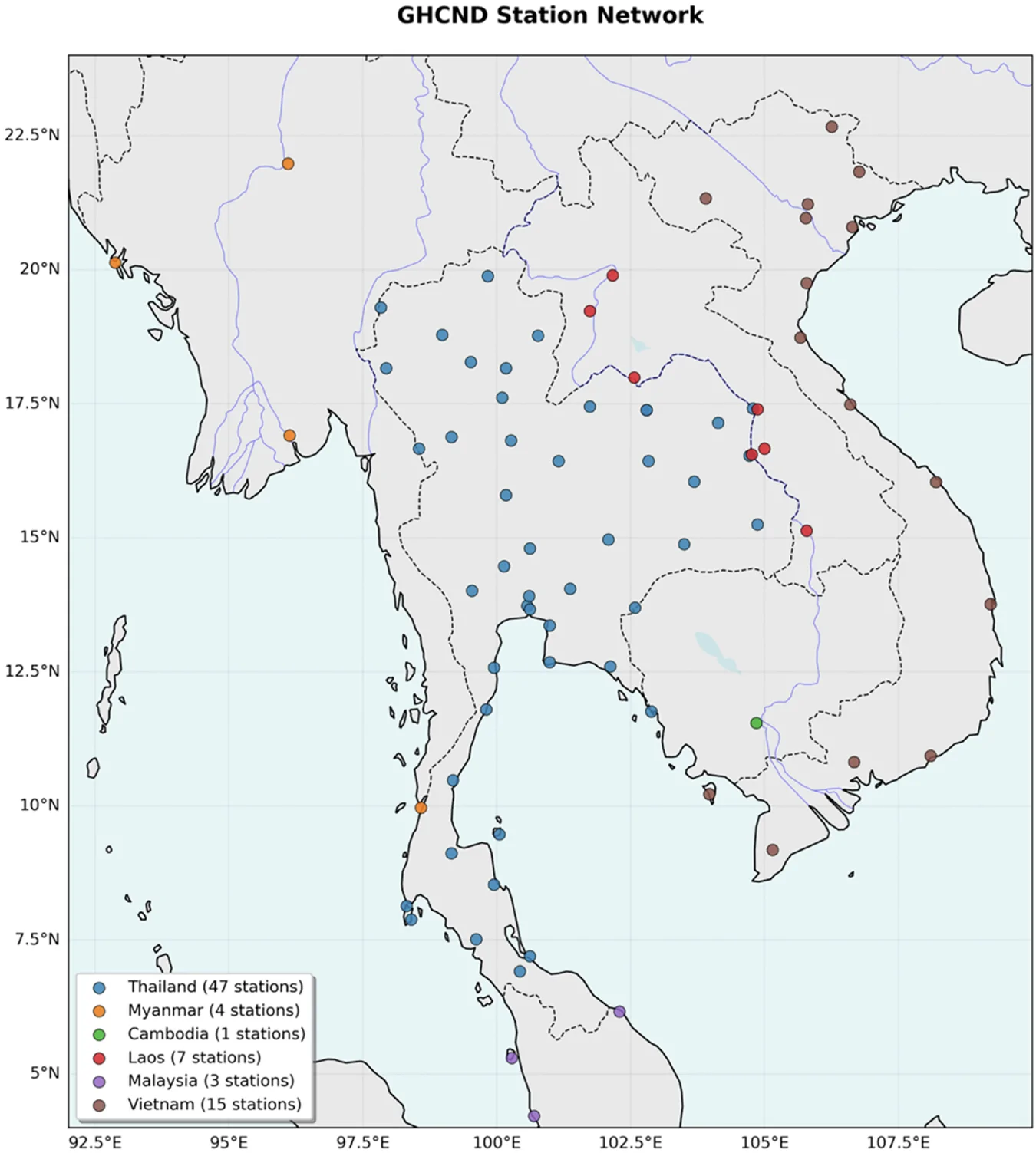
Study area and location of GHCND station network.

The 77 GHCND stations used for independent validation are summarized in Table 1 and mapped in Fig. 1; full background on the GHCND database itself, its 19 automated QC checks, and its known limitations for tropical Southeast Asia is given in Data Sources subsection. The validation network is dominated by lowland sites — 65 of the 77 stations lie below 200 m, 11 between 200 and 500 m, and only one station (Son La, Vietnam, 676 m) falls within the 500–1000 m band, yielding a median elevation of 35 m and a maximum of 676 m. No station in the validation set exceeds 1000 m. Consequently, the highland belts of the Arakan Yoma, Chin Hills, northern Laos, and the Annamites above 1000 m remain entirely unsampled by the validation network^42^, and the 500–1000 m band is represented by a single station. The validation metrics are therefore interpreted as indicative of MSEA-Daily performance in lowland, populated environments rather than as fully representative of the entire domain.

Each station was paired with its containing TerraClimate grid cell for direct comparison. Evaluation metrics — bias (mean difference), mean absolute error (MAE), root mean square error (RMSE), Pearson correlation (r), Nash–Sutcliffe Efficiency (NSE) and Kling–Gupta Efficiency (KGE) — were computed per station and then aggregated across stations using equal weighting to avoid spatial-clustering bias (Hypothesis A; full metric definitions in Supplementary Methods S3). For precipitation, r, RMSE, MAE, bias, NSE and KGE were computed on all days of the overlapping station–grid record (including dry days at both sides). All-days metrics were retained as the primary statistic because dry-day misclassification (false-positive or false-negative wet days) is itself a relevant skill component for hydrological and agricultural applications such as crop water-balance modelling, consecutive-dry-day indices and water-deficit accounting; restricting evaluation to wet days would mask this component of dataset performance. For temperature, NSE_anomaly and Pearson r were additionally computed on deseasonalised daily anomalies to isolate day-to-day variability skill independent of the seasonal cycle.

### Independent validation against GHCND stations

Table S1 reports validation metrics for MSEA-Daily daily fields against GHCND station observations located within the extended product domain (4–24 °N, 92–110 °E) during 1981–2014. Of the 77 in-domain GHCND stations, all 77 satisfied the precipitation QC filter and 68/66/67 contributed to Tmax/Tmin/Tmean. Averaged across the whole in-domain network, precipitation exhibits r = 0.61, KGE = 0.36 and NSE = 0.30, with a small dry bias of −0.96 mm day^−1^, MAE of 1.51 mm day^−1^ and RMSE of 3.25 mm day^−1^; maximum temperature shows r = 0.96, KGE = 0.92, NSE = 0.88 (bias −0.40 °C, MAE 0.51 °C, RMSE 0.66 °C); minimum temperature achieves r = 0.94, KGE = 0.94, NSE = 0.86 (bias −0.28 °C, MAE 0.52 °C, RMSE 0.63 °C); and mean temperature gives r = 0.97, KGE = 0.95, NSE = 0.93 (bias −0.02 °C, MAE 0.29 °C, RMSE 0.42 °C). A spatially explicit per-station view of these metrics is provided in Supplementary Figure S1, where each of the 77 stations is colour-coded by its daily-resolution KGE for each of the four variables.

The stratified rows of Table S1 report metrics by country (Thailand n = 47, Vietnam n = 15, Laos n = 7, Myanmar n = 4, Malaysia n = 3, Cambodia n = 1), elevation band, and coastal/inland class (n = 34 coastal, n = 43 inland). Country-level metrics for Thailand and Vietnam are reported without qualifier as both subsets exceed the n ≥ 10 robustness threshold; metrics for Laos, Myanmar, Malaysia, and Cambodia are flagged † (indicative, n < 10) or ‡ (single-station daily fallback at n = 1 for Cambodia) as documented in the table footnote. The validation network is heavily concentrated below 200 m elevation (65 of 77 stations), with only one station between 500 and 1000 m and none above 1000 m, so stratum-level metrics for highland and other complex-topography environments within the domain are not directly constrained by this validation.

**Fig. 2:**
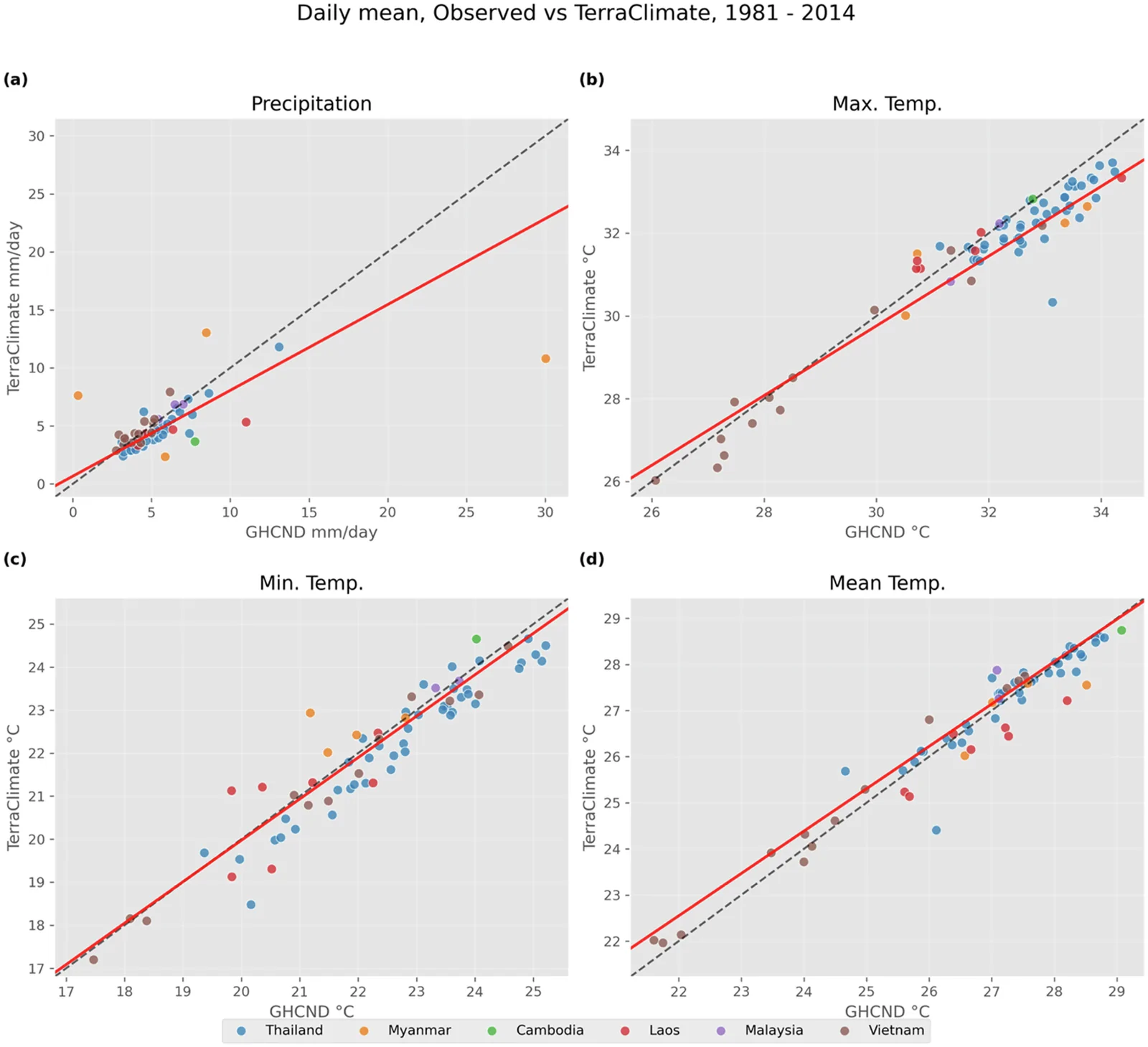
Independent validation against GHCND stations: scatterplots comparing MSEA-Daily with daily station observations for (**a**) precipitation, (**b**) maximum temperature, (**c**) minimum temperature, and (**d**) mean temperature during 1981–2014 across mainland Southeast Asia.

Across all four variables (Fig. 2), the scatter is concentrated along the 1:1 line: the temperature panels (b–d) cluster tightly with regression slopes indistinguishable from unity and a small consistent cool offset of order −0.4 °C in Tmax, while precipitation (a) shows wider scatter — particularly above 10 mm day^−1^ — with one outlier station whose observed climatology of ≈30 mm day^−1^ is reproduced by MSEA-Daily as ≈11 mm day^−1^.

**Fig. 3:**
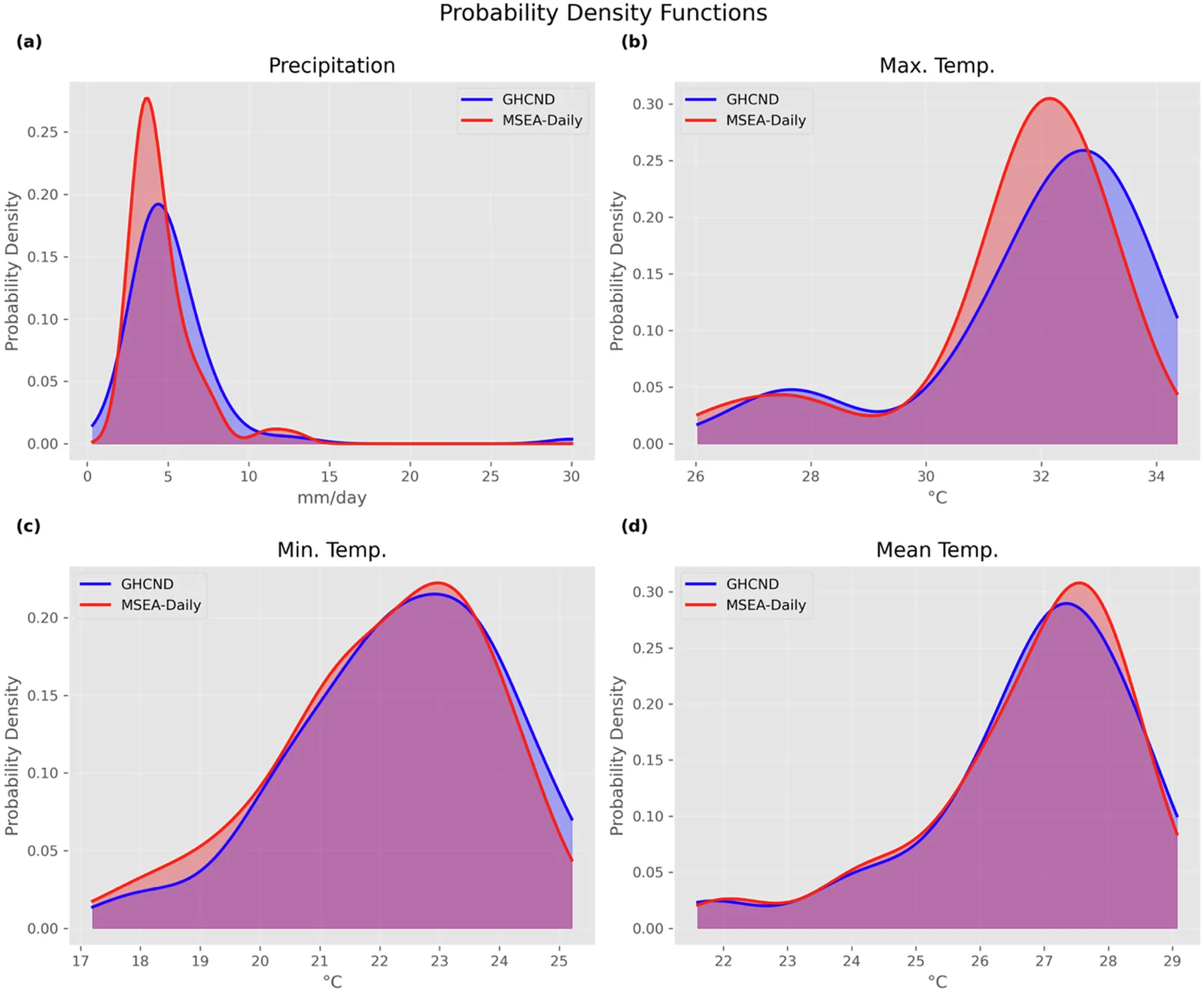
Probability density functions comparing MSEA-Daily with GHCND station observations for (**a**) precipitation, (**b**) maximum temperature, (**c**) minimum temperature, and (**d**) mean temperature during 1981–2014.

The MSEA-Daily and GHCND distributions overlap closely for the three temperature variables (Fig. 3b–d), with peaks within ≈0.5–1 °C and density tails matching across the 17–34 °C range; the largest distributional offset is in the precipitation panel (Fig. 3a), where MSEA-Daily has a sharper, taller peak at ≈4 mm day^−1^ and a steeper probability decline above ≈7 mm day^−1^, indicating under-representation of intense daily rainfall events at the upper-tail end. The four-season decomposition in Figure S2 confirms that these distributional features persist across all four meteorological seasons, and higher-order distributional statistics (Kolmogorov–Smirnov statistic, skewness difference, excess-kurtosis difference, and KS p-values) are reported per station in Figure S3.

**Fig. 4:**
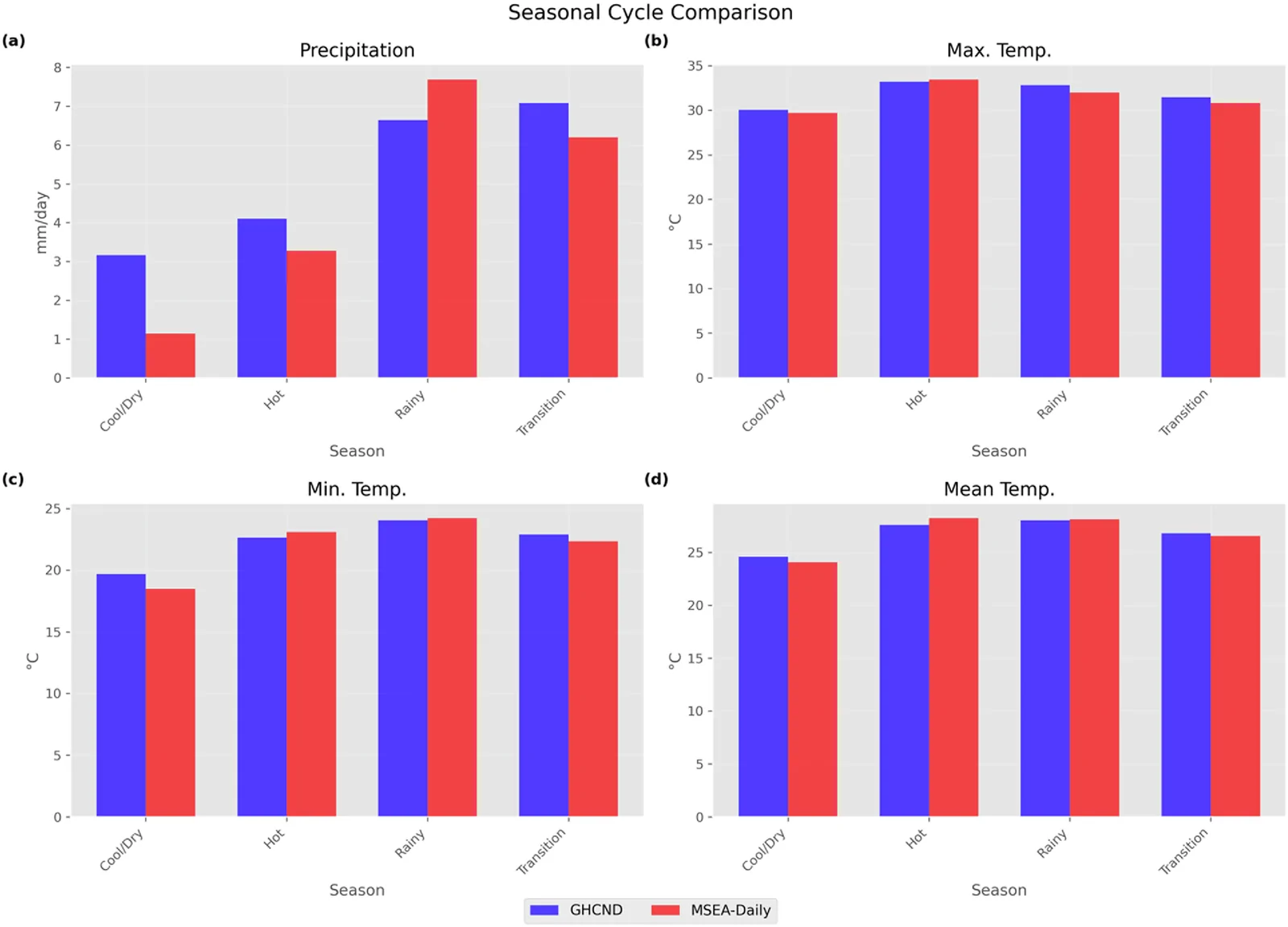
Seasonal cycle comparison between GHCND station observations and MSEA-Daily monthly climatology for (**a**) precipitation, (**b**) maximum temperature, (**c**) minimum temperature, and (**d**) mean temperature across four meteorological seasons: Cool/Dry (December–February, DJF), Hot (March–May, MAM), Rainy (June–August, JJA), and Transition (September–November, SON) during 1981–2014.

The seasonal-cycle comparison is shown in Fig. 4. Seasonally, MSEA-Daily reproduces GHCND mean climatologies within ≈ ± 0.4 °C for Tmax and Tmean during all four seasons, except a cool offset of ≈ −0.8 °C for Tmax in JJA and SON; the largest seasonal disagreement is a precipitation dry bias of ≈ −2.1 mm day^−1^ (about −65% of the observed value) in the cool/dry season DJF, with smaller seasonal precipitation biases of ≈ −0.9, +1.0, and −0.9 mm day^−1^ in MAM, JJA and SON respectively. Tmin disagrees by ≈ −1.2 °C in DJF, agrees within ±0.4 °C in MAM and JJA, and is ≈ −0.6 °C below GHCND in SON.

**Fig. 5:**
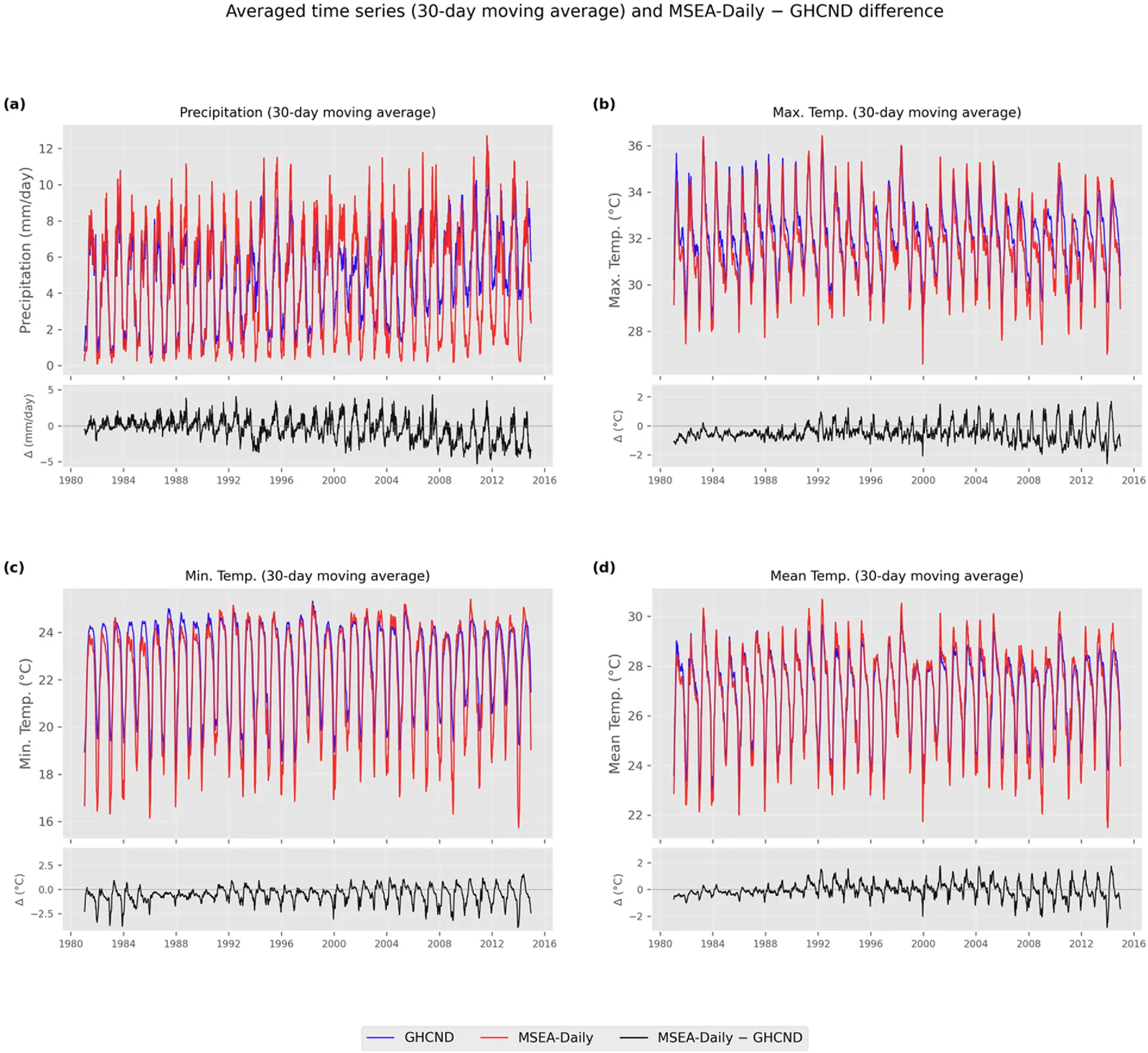
Averaged time series comparing GHCND station observations and MSEA-Daily monthly climatology with 30-day moving averages for (**a**) precipitation, (**b**) maximum temperature, (**c**) minimum temperature, and (**d**) mean temperature during 1981–2014 across mainland Southeast Asia. In each panel, the upper sub-panel shows the GHCND observed (blue) and MSEA-Daily (red) 30-day moving averages, and the lower sub-panel shows the difference MSEA-Daily − GHCND (black) on a separate y-axis with a horizontal zero-reference line.

The two series track each other tightly across the 1981–2014 record for all four variables (Fig. 5), with the most consistent disagreement being the dry-season precipitation bias visible in the Fig. 5a difference sub-panel. The difference sub-panels (lower row of each panel) confirm that the residual MSEA-Daily − GHCND magnitudes are temporally stationary across 1981–2014, consistent with the domain-averaged biases reported in Table S1; no systematic drift in the residual is visible across the 34-year record.

### Source-data benchmarking

**Fig. 6:**
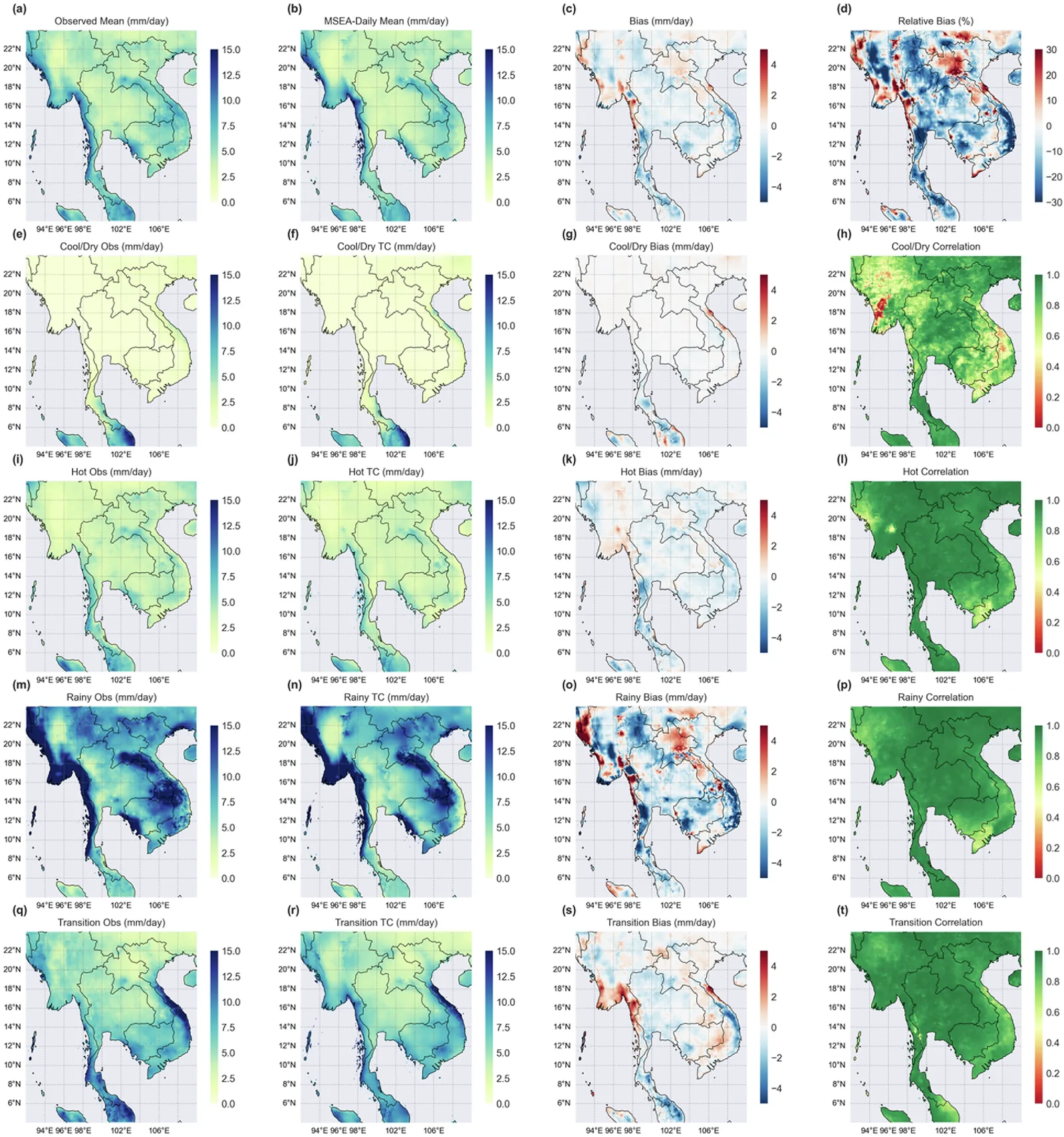
Spatial precipitation patterns and biases (1981–2014) (**a**) CHIRPS annual mean, (**b**) MSEA-Daily annual mean, (**c**) absolute bias, (**d**) relative bias (%), (**e,f**) cool/dry season observations and model, (**g,h**) cool/dry season bias and correlation, (**I,j**) hot season observations and model, (k-l) hot season bias and correlation, (m-n) rainy season observations and model, (**o,p**) rainy season bias and correlation, (**q,r**) transition season observations and model, (s-t) transition season bias and correlation.

Table S2 and Figure S4 report the corresponding CHIRPS–GHCND (precipitation) and AgERA5–GHCND (Tmax/Tmin/Tmean) metrics across the same 77 stations and the same Table S1 stratification, so MSEA-Daily–GHCND skill (Table S1) can be read row-for-row against the source-dataset–GHCND skill of its two upstream products. Precipitation: MSEA-Daily inherits CHIRPS’s spatial-skill structure essentially unchanged. Domain-average across-station precipitation skill is virtually identical between MSEA-Daily and CHIRPS (r = 0.61 vs 0.65; KGE = 0.36 vs 0.38; RMSE = 3.25 vs 3.12 mm day^−1^; bias = −0.96 vs −0.87 mm day^−1^), and the same near-identical pattern holds across every country, elevation, and coastal stratum in Table S1. This confirms that the monthly-conservation disaggregation preserves CHIRPS’s daily spatial-pattern skill faithfully — the biases and skill structure of MSEA-Daily precipitation are inherited from CHIRPS rather than introduced or amplified by the disaggregation step. The light-rain over-frequency at 2–5 mm day^−1^ in Fig. 3a, the Chin-Arakan windward over-estimation in Fig. 6, and the heavy-rainfall under-representation are accordingly CHIRPS features inherited by MSEA-Daily, consistent with the documented CHIRPS limitations cited. Temperature: MSEA-Daily substantially mitigates AgERA5’s systematic biases. The most striking result is that MSEA-Daily Tmax skill against GHCND is markedly better than raw AgERA5 Tmax skill against the same stations (r = 0.96 vs 0.91; RMSE = 0.66 vs 2.20 °C; NSE = 0.88 vs −0.36; bias = −0.40 vs −2.05 °C); the −2.05 °C AgERA5 cool bias against GHCND is reduced to −0.40 °C in MSEA-Daily. The same pattern holds for Tmean (r = 0.97 vs 0.94; RMSE = 0.42 vs 1.30 °C; bias = −0.02 vs −1.16 °C) and to a smaller degree for Tmin (r = 0.94 vs 0.92; RMSE = 0.63 vs 0.79 °C). This mitigation is the expected outcome of the TerraClimate-anchored disaggregation: the daily-anomaly addition preserves AgERA5’s temporal variability structure (correlation skill carries through and slightly improves) while the TerraClimate monthly-mean climatological target replaces AgERA5’s reanalysis-derived monthly mean with a station-gridded one, eliminating most of the AgERA5 cool bias against *in-situ* observations. This is the inverse of an “amplification” outcome — MSEA-Daily delivers temperature skill superior to its primary daily source by combining AgERA5’s day-to-day variability with TerraClimate’s better-anchored monthly means.

### Internal consistency verification

Table 2 presents internal consistency metrics comparing MSEA-Daily against source datasets. Precipitation versus CHIRPS shows r = 0.88, KGE = 0.80, NSE = 0.71, with a small dry bias of −0.33 mm day^−1^, MAE of 1.71 mm day^−1^, and RMSE of 6.31 mm day^−1^. Maximum temperature versus AgERA5 shows r = 0.94, KGE = 0.93, NSE = 0.81, with a warm bias of +1.23 °C, MAE of 1.64 °C, and RMSE of 1.97 °C. Minimum temperature versus AgERA5 shows r = 0.96, KGE = 0.92, NSE = 0.91, with a small cool bias of −0.46 °C, MAE of 1.05 °C, and RMSE of 1.41 °C. Mean temperature versus AgERA5 shows r = 0.96, KGE = 0.93, NSE = 0.85, with a warm bias of +1.15 °C, MAE of 1.37 °C, and RMSE of 1.63 °C. Notably, the Tmean warm bias of +1.15 °C is markedly larger than the + 0.39 °C that would be expected from a simple (Tmax + Tmin)/2 combination of the Tmax (+1.23 °C) and Tmin (−0.46 °C) biases. These error magnitudes are expected outcomes of the disaggregation procedure rather than dataset accuracy statistics. The precipitation RMSE of 6.31 mm day^−1^ quantifies the deliberate daily redistribution performed under the strict monthly-conservation constraint: MSEA-Daily preserves the CHIRPS-derived monthly total exactly while reshaping individual daily values via the α = 1.35 power transform, so daily values necessarily diverge from the unscaled CHIRPS daily field by construction. The + 1.23 °C Tmax bias against AgERA5 reflects the additive monthly-mean offset between TerraClimate, which serves as MSEA-Daily’s monthly target, and AgERA5, which contributes only the zero-mean daily anomalies added to that target; because each daily MSEA-Daily Tmax value is generated by superimposing the AgERA5 daily anomaly onto the corresponding TerraClimate monthly mean, the bias of MSEA-Daily relative to AgERA5 inherits the climatological-mean difference between the two reference fields directly and mechanically. The + 1.15 °C Tmean bias is the analogous offset between the TerraClimate monthly mean and AgERA5’s 24-hour-mean Tmean, with the additional diurnal-midpoint-versus-24-hour-mean asymmetry already discussed above explaining why it does not simply equal the average of the Tmax and Tmin biases. Within Table 2, r, NSE and KGE are the appropriate internal-consistency indicators because they capture temporal-pattern preservation, while bias and RMSE quantify the expected redistribution and reference-field offsets.

**Table 2:** Internal consistency metrics comparing MSEA-Daily against source datasets.

Variable	vs Dataset	RMSE	MAE	Bias	Pearson r	NSE	KGE
Precipitation	CHIRPS	6.31	1.71	−0.33	0.88	0.71	0.80
Maximum Temp	AgERA5	1.97	1.64	+1.23	0.94	0.81	0.93
Minimum Temp	AgERA5	1.41	1.05	−0.46	0.96	0.91	0.92
Mean Temp	AgERA5	1.63	1.37	+1.15	0.96	0.85	0.93

Figure S5 shows close pattern agreement throughout the seasonal cycle and across the percentile distribution, with the small −0.33 mm day^−1^ bias arising chiefly from the power-transformation step used to disaggregate monthly TerraClimate totals into daily fields. Figure S6 (maximum temperature versus AgERA5) shows that the +1.23 °C warm bias is broadly seasonally uniform with a small additional expansion in the pre-monsoon hot months when AgERA5 itself reaches its annual maximum. Figure S7 (mean temperature versus AgERA5) shows the +1.15 °C warm bias maintained across all six diagnostic dimensions and across all four seasons, consistent with a reference-field-definition offset (24-hour mean vs. diurnal midpoint) rather than a defect in the downscaling procedure itself. Figure S8 (minimum temperature versus AgERA5) shows tight climatological agreement, with the small −0.46 °C cool bias concentrated in cool-season nocturnal minima.

### Spatial validation patterns

At annual scale, MSEA-Daily reproduces the CHIRPS spatial pattern with point-wise spatial correlation of r ≈ 0.95 (Fig. 6a vs 6b); absolute biases (Fig. 6c) are generally below 2 mm day^−1^ except for a positive belt of + 3 to + 5 mm day^−1^ along the northwestern Myanmar/Chin-Arakan windward slope, and the relative-bias map (Fig. 6d) shows MSEA-Daily underestimating CHIRPS by 10–25% across most of the interior of the domain. Seasonal panels (Fig. 6e–t and Supplementary Figures S9–S10) show small biases and high spatial correlations (r ≥ 0.7 across nearly the entire domain) in all four seasons, with the largest seasonal absolute biases occurring in the rainy season (JJA) along the Chin-Arakan slope and the smallest in the cool/dry season (DJF) where domain-wide precipitation totals are below 3 mm day^−1^.

**Fig. 7:**
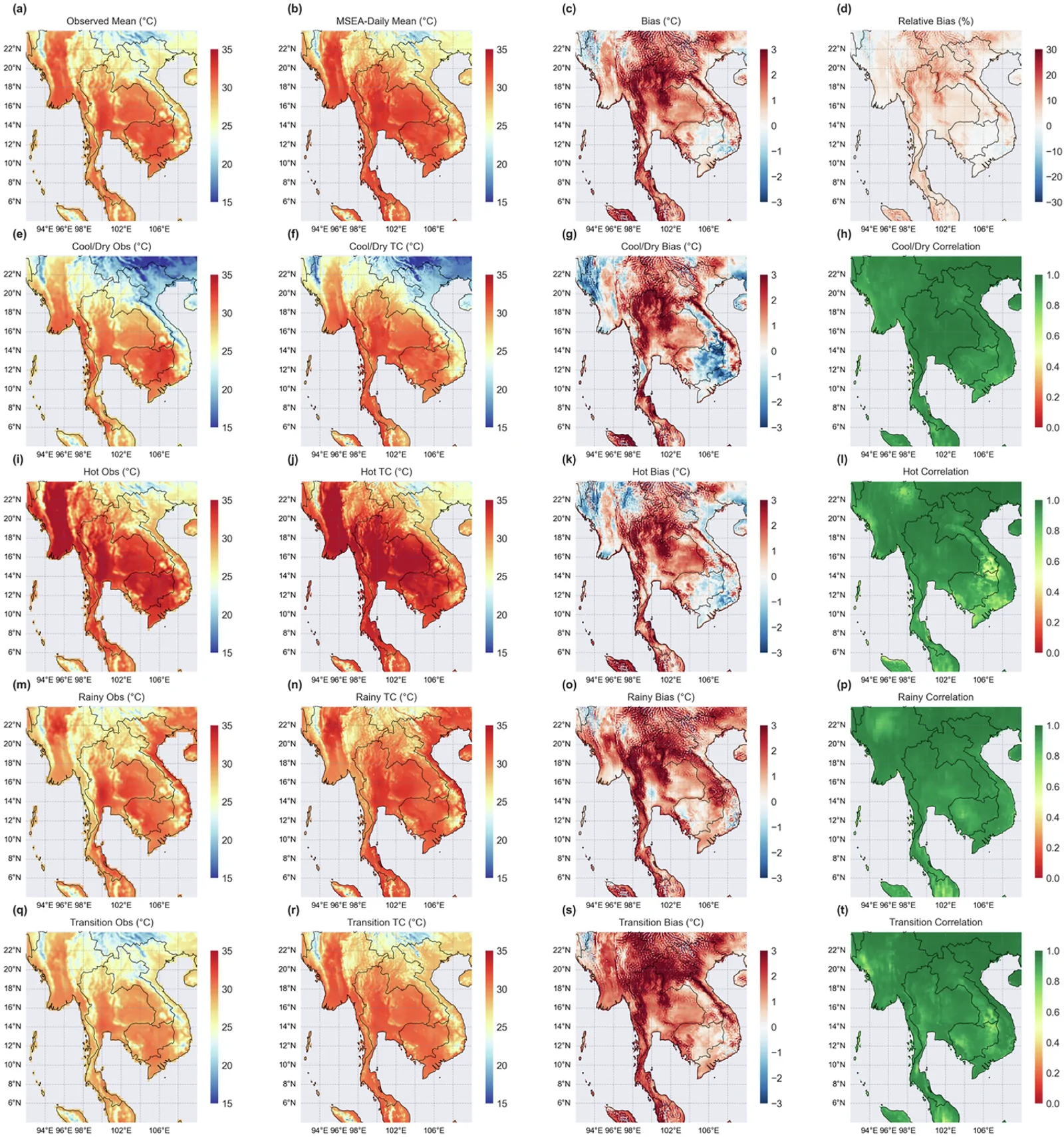
Spatial maximum temperature patterns and biases (1981–2014) (**a**) AgERA5 annual mean, (**b**) MSEA-Daily annual mean, (**c**) absolute bias, (**d**) relative bias (%), (**e-f**) cool/dry season observations and model, (**g-h**) cool/dry season bias and correlation, (**i-j**) hot season observations and model, (**k-l**) hot season bias and correlation, (m-n) rainy season observations and model, (**o-p**) rainy season bias and correlation, (**q-r**) transition season observations and model, (s-t) transition season bias and correlation.

MSEA-Daily reproduces the AgERA5 Tmax annual climatology at first order (Fig. 7a,b), with point-wise spatial correlation of r ≈ 0.95 and faithful preservation of the elevation lapse rate. The annual absolute bias (Fig. 7c) is +1 to +2 °C across most of the domain, with the largest residuals (+2 to +3 °C) along the Chin-Arakan flank, corresponding to +5 to +10% of climatological Tmax (Fig. 7d) and consistent with the +1.23 °C internal-consistency bias against AgERA5 reported in Table 2. Seasonally, the bias is most spatially structured in the cool/dry season (Fig. 7e–h, Figures S11–S12), which shows a positive-bias/negative-bias dipole between the Chin-Arakan and northern-Vietnamese highlands (+2 to + 3 °C) and the southern-Vietnam/Mekong-Delta/Gulf-of-Thailand coastal lowlands (−1 to −3 °C); the hot, rainy and transition seasons (Fig. 7i–t) show smaller and more spatially uniform biases, mostly within ±1 °C, with the residual positive anomaly persisting along the Chin-Arakan flank. Spatial correlations remain uniformly high (r ≈ 0.8–1.0) across all four seasons.

**Fig. 8:**
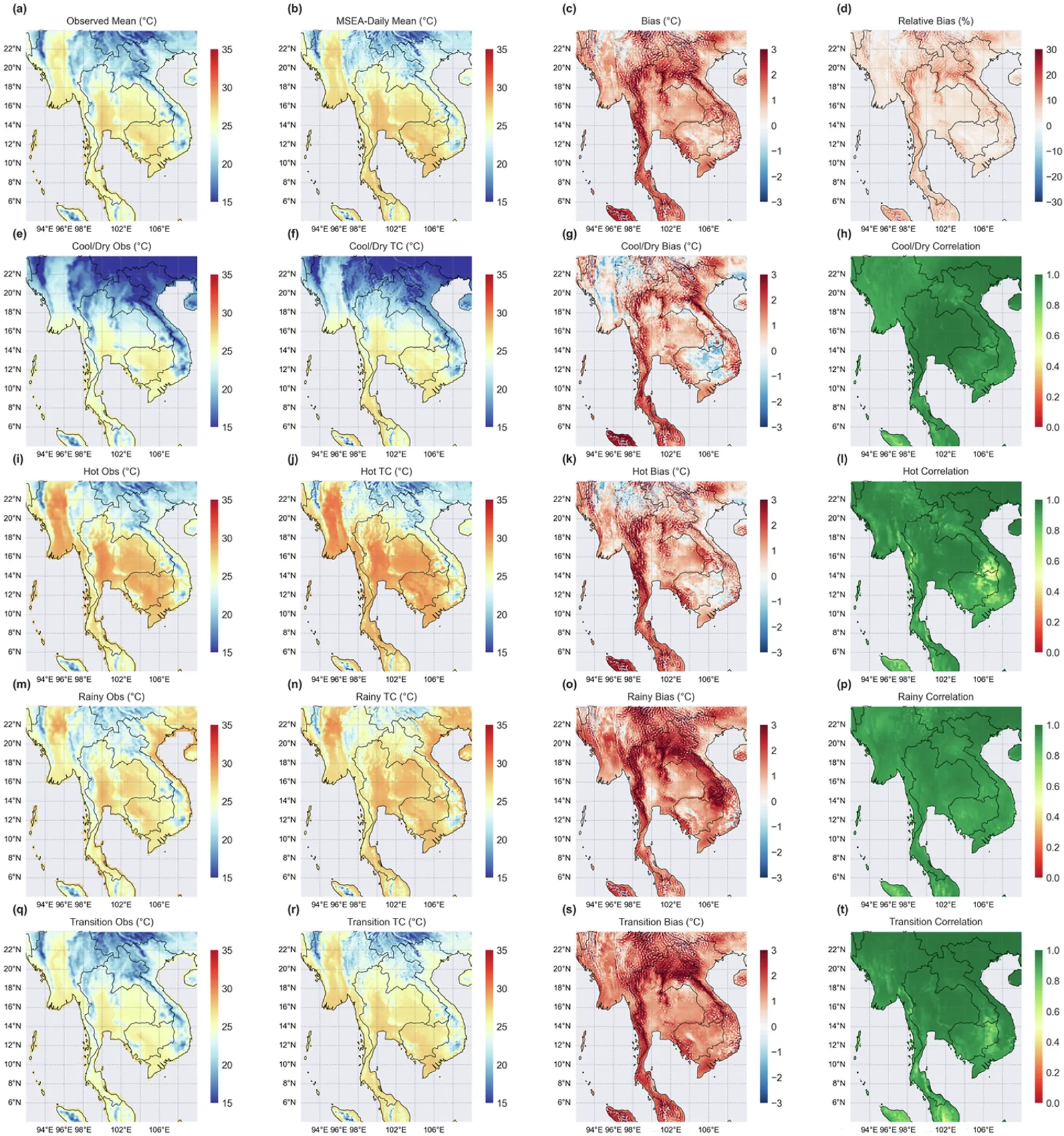
Spatial mean temperature patterns and biases (1981–2014) (**a**) AgERA5 annual mean, (**b**) MSEA-Daily annual mean, (**c**) absolute bias, (**d**) relative bias (%), (**e-f**) cool/dry season observations and model, (**g-h**) cool/dry season bias and correlation, (**i-j**) hot season observations and model, (**k-l**) hot season bias and correlation, (m-n) rainy season observations and model, (**o-p**) rainy season bias and correlation, (**q-r**) transition season observations and model, (s-t) transition season bias and correlation.

MSEA-Daily reproduces the AgERA5 Tmean annual climatology at first order (Fig. 8a,b), with point-wise spatial correlation of r ≈ 0.97 and faithful preservation of the elevation lapse rate. The annual absolute bias (Fig. 8c) is +1 to +2 °C across most of the domain, with the strongest positive residuals (+2 to +3 °C) over the Chin-Arakan, northern-Lao and northern-Vietnamese highlands, broadly consistent with the +1.15 °C internal-consistency bias against AgERA5 reported in Table 2. Seasonally (Fig. 8e–t, Figures S13–S14), the Tmean bias pattern is more spatially uniform than the Tmax case and lacks the cool/dry-season coastal dipole; biases are smallest in DJF (mostly +0.5 to +1 °C) and somewhat larger but elevation-keyed (+1 to +2 °C, with +2 to +3 °C lobes over the highlands) in MAM, JJA and SON. Spatial correlations remain uniformly high (r ≈ 0.9–1.0) across all four seasons.

**Fig. 9:**
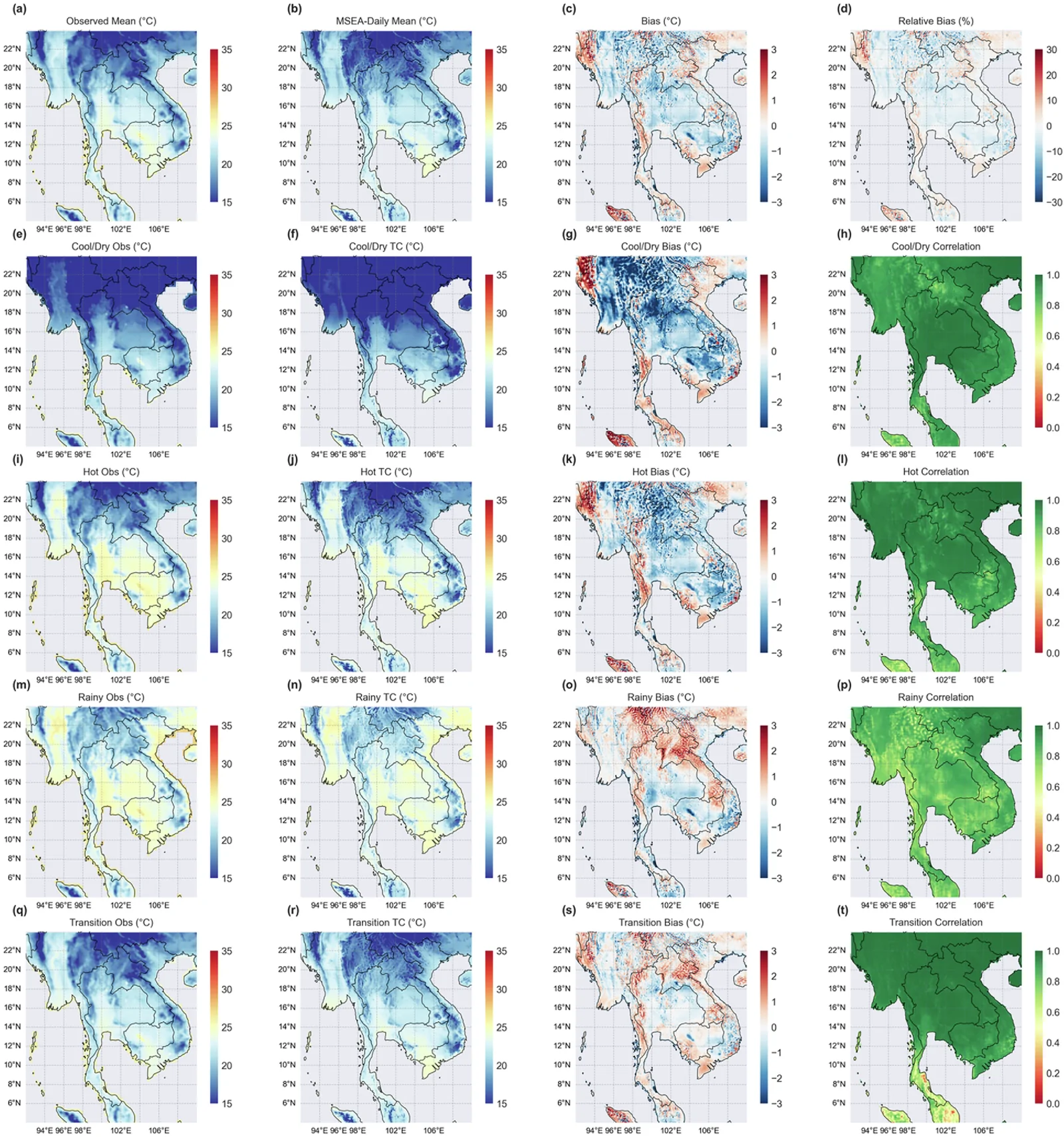
Spatial minimum temperature patterns and biases (1981–2014) (**a**) AgERA5 annual mean, (**b**) MSEA-Daily annual mean, (**c**) absolute bias, (**d**) relative bias (%), (**e-f)** cool/dry season observations and model, (**g-h**) cool/dry season bias and correlation, (**i-j**) hot season observations and model, (**k-l**) hot season bias and correlation, (m-n) rainy season observations and model, (**o-p**) rainy season bias and correlation, (**q-r**) transition season observations and model, (**s-t**) transition season bias and correlation.

MSEA-Daily reproduces the AgERA5 Tmin annual climatology at first order (Fig. 9a,b), with point-wise spatial correlation of r ≈ 0.96. The annual absolute bias (Fig. 9c) is predominantly small (typically within ± 1 °C) with mixed sign across the domain, consistent with the small −0.46 °C internal-consistency bias against AgERA5 reported in Table 2. The dominant seasonal feature is a cool/dry-season dipole (Fig. 9e–h, Figures S15–S16): a positive bias of +2 to +3 °C over the Chin-Arakan slope, contrasted with a coherent negative bias of −1 to −3 °C across most of Vietnam, eastern Laos and the northeastern Thai interior. The hot, rainy and transition seasons show much smaller biases (mostly within ± 1 °C). Spatial correlations remain uniformly high (r ≈ 0.9–1.0) across all four seasons.

### Temporal dynamics

Time-latitude (Hovmöller) diagrams compare the 1981–2014 evolution of MSEA-Daily and the upstream source dataset for each variable: precipitation (Figure S17, MSEA-Daily vs CHIRPS), maximum temperature (Figure S18, MSEA-Daily vs AgERA5), mean temperature (Figure S19, MSEA-Daily vs AgERA5), and minimum temperature (Figure S20, MSEA-Daily vs AgERA5). Each diagram has three panels: source dataset, MSEA-Daily, and the bias field MSEA-Daily − source.

In all four diagrams, the MSEA-Daily and source-dataset panels are visually indistinguishable at the panel scale — the seasonal-monsoon precipitation bands, the pre-monsoon Tmax warming, the seasonal Tmin cycle, and the meridional gradients all overlap year-on-year, confirming that the disaggregation preserves the temporal evolution of the upstream source dataset at decadal scale. The bias fields (Figures S17c, S18c, S19c, S20c) are dominated by small-magnitude high-frequency residuals consistent with the internal-consistency biases reported in Table 2 (precipitation −0.33 mm day^−1^; Tmax + 1.23 °C; Tmean + 1.15 °C; Tmin −0.46 °C); none of the four variables shows a long-term drift in the bias across the 34-year record, indicating that the bias structure is temporally stationary at decadal scale and that the dataset is suitable for trend-analysis applications without time-dependent bias correction.

### Preservation of interannual variability

**Fig. 10:**
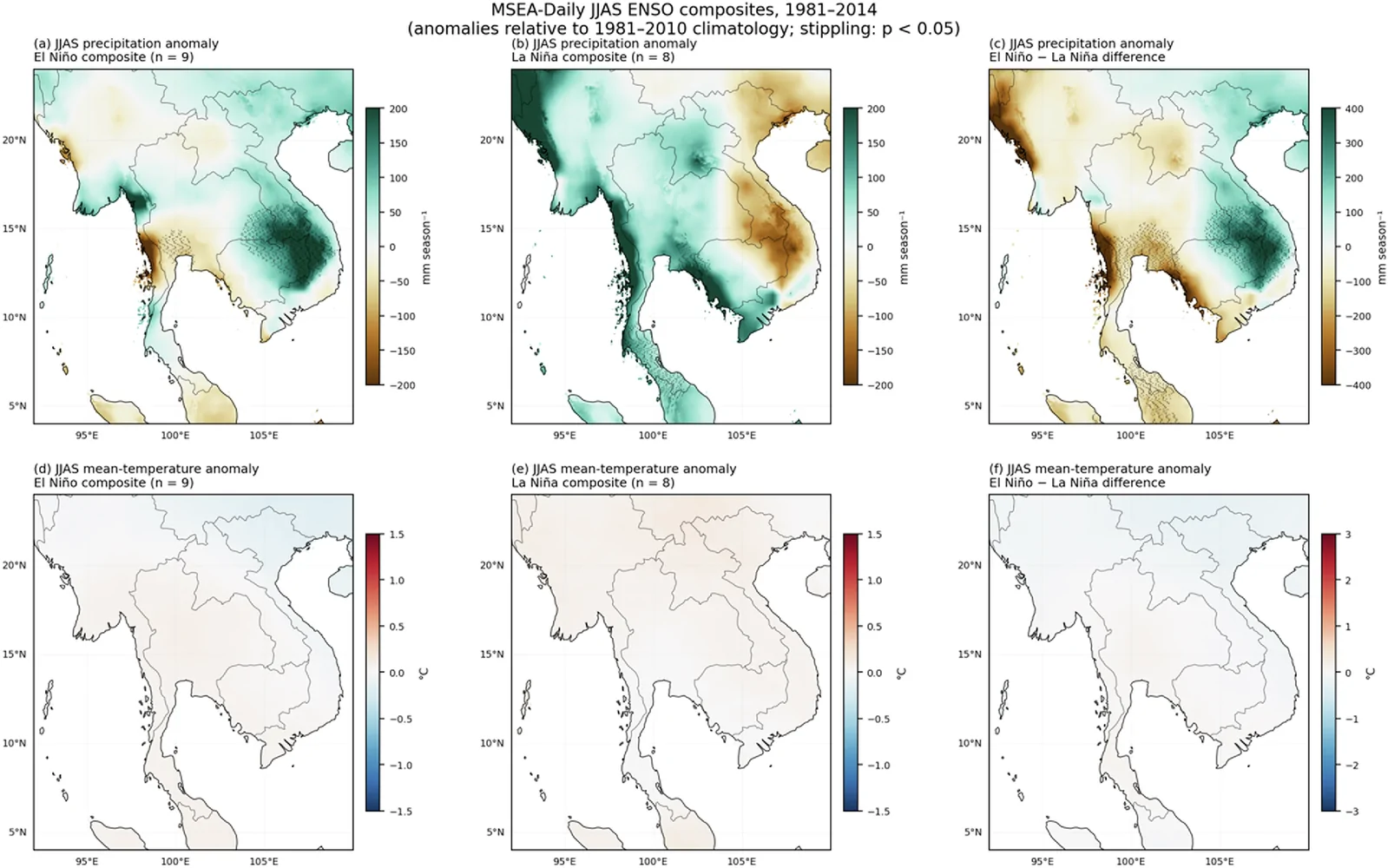
JJAS ENSO composites of MSEA-Daily fields, 1981–2014. Top row (**a–c**): precipitation anomalies (mm season^−1^). Bottom row (**d–f**): mean-temperature anomalies (°C).

As a final quality check, we verified that the temporal disaggregation preserves the interannual variability of the upstream source datasets without measurable distortion. Using NOAA’s Oceanic Niño Index (ONI; Niño 3.4 SST anomaly, 1981–2014) as a well-characterised driver of regional interannual variability over mainland Southeast Asia, we computed per-pixel correlation between the JJAS-aggregated MSEA-Daily anomaly fields and the JJAS ONI, and compared these correlation patterns to the same calculation applied to the upstream CHIRPS (precipitation) and AgERA5 (mean temperature) source datasets. The MSEA-Daily and CHIRPS correlation maps for precipitation, and the MSEA-Daily and AgERA5 correlation maps for mean temperature, are spatially indistinguishable at the same colour scale (Figure S21), confirming that the disaggregation transmits the interannual variability of the upstream sources to the daily MSEA-Daily fields without measurable distortion. The composite anomaly maps (Fig. 10) and supporting time-series, lead-lag, and scatter analyses (Figures S22–S24, Table S3) are provided in the Supplementary Material so that users planning interannual or ENSO-related applications can inspect MSEA-Daily’s behaviour against widely documented expectations; the Validation Considerations paragraph in Usage Notes summarises MSEA-Daily’s suitability for these applications.

## Usage Notes

### Systematic biases and corrections

The systematic biases of MSEA-Daily relative to GHCND station observations are spatially heterogeneous across mainland Southeast Asia and cannot be removed by a single uniform additive or multiplicative correction. Users requiring bias-corrected fields should therefore consult the stratified independent-validation results in Table S1 (per country, elevation band, and coastal/inland class) and the spatial bias maps in Figs. 6–9 to identify the bias structure relevant to their domain of interest, and apply station-anchored quantile mapping locally where station data are available. The summary that follows is therefore framed as illustrative, region-dependent guidance rather than as prescriptive whole-domain adjustments.

### Domain-average context (against GHCND)

Maximum temperature has a small mean cool bias of −0.40 °C (per-station spread approximately ±0.7 °C across the 77-station network), minimum temperature −0.28 °C (±0.6 °C), mean temperature −0.02 °C (±0.4 °C), and precipitation a domain-mean dry bias of −0.96 mm day^−1^ (per-station spread of order ±2 mm day^−1^). These domain-average biases are an order of magnitude smaller than the corresponding biases of the source datasets (AgERA5 Tmax bias against GHCND is −2.05 °C, AgERA5 Tmean bias is −1.16 °C; see Table S2), reflecting the bias-mitigation effect of the TerraClimate-anchored disaggregation chain documented in Source-Data Benchmarking subsection. Users should not, however, infer from these domain averages that a uniform additive correction is appropriate.

### Spatial heterogeneity (against AgERA5)

Internal consistency against AgERA5 shows additive offsets of order +1 to +3 °C concentrated over the Chin-Arakan slope and the northern-Lao/northern-Vietnamese highlands, near-zero offsets over the lowland interior, and an opposite-sign cool/dry-season Tmin dipole over Vietnam and the Mekong Delta (Fig. 9g). Precipitation biases against CHIRPS are negligible by construction in the climatological mean and largely localised to the windward Chin-Arakan slope (Fig. 6c). The spatial structure documented in Figs. 6–9 should be the primary reference for any region-specific bias adjustment, not a single domain-average factor.

### Frequency and extreme indices

Frequency-based precipitation indices (wet-day count, consecutive dry/wet days, R10mm, R20mm, simple-daily-intensity index SDII) inherit the inflated 2–5 mm day^−1^ peak visible in Fig. 3a and consequently over-count wet days at light-rain thresholds; users computing such indices should apply a station-calibrated trace-event threshold (typically 1 mm day^−1^ rather than the conventional 0.1 mm day^−1^) or use CHIRPS directly when the application requires only frequency information. Indices targeting warm extremes (TX90p, TXx, hot-day count above 35 °C, warm-spell-duration index) inherit AgERA5’s damping of the upper Tmax tail visible in Fig. 3b, so MSEA-Daily under-represents the frequency of days exceeding 34 °C by ~10–20% relative to GHCND; for heat-wave applications, users should apply station-anchored quantile mapping locally rather than a uniform multiplicative correction. The previous-version recommendation of multiplicative factors of 1.12 (95th percentile) and 1.20 (99th percentile) for extreme precipitation should be regarded as an illustrative central tendency only — the actual under-representation varies between approximately 5% and 30% across stations (CHIRPS-inherited; see Funk *et al*.^9^) and is best handled by per-station quantile mapping where local observations exist.

**Monthly-scale analyses**. Match TerraClimate exactly by construction and require no correction. The temporal disaggregation conserves TerraClimate’s monthly precipitation totals and monthly temperature means at every grid cell, so MSEA-Daily aggregated to monthly resolution is mathematically identical to TerraClimate. As a consequence, the daily MSEA-Daily-vs-GHCND independent validation, when interpreted at monthly aggregation, also serves as a direct in-region validation of TerraClimate against the 77-station mainland Southeast Asia network for the 1981–2014 study period. This in-region evidence confirms that TerraClimate is sufficiently reliable to serve as the monthly anchor of MSEA-Daily across mainland Southeast Asia. Users running monthly-scale impact assessments with MSEA-Daily can therefore do so with confidence equivalent to using TerraClimate directly

#### Appropriate and inappropriate uses

MSEA-Daily is appropriate for applications requiring consistency between monthly climatology (trends) and daily data (process modeling), including agricultural water balance models using TerraClimate for long-term planning with daily crop simulations, hydrological models requiring daily inputs while maintaining monthly water balance consistency, and climate indices (consecutive dry days, heat wave duration) where monthly context must be preserved. The dataset is not appropriate for applications focused on precipitation extremes, rainfall-frequency indices, or maximum-temperature extremes without applying corrections, real-time monitoring or forecasting (data ends 2014), studies requiring sub-daily temporal resolution, analysis of diurnal temperature cycles, or applications where maximum temperature absolute accuracy is critical without correction. Users should use CHIRPS directly for precipitation-only applications not requiring temperature variables or TerraClimate consistency. AgERA5 should be used when sub-daily resolution is required, comprehensive meteorological variables beyond precipitation and temperature are needed, or pre-1981 data is necessary.

#### Geographic and temporal limitations

Accuracy is expected to be reduced in complex terrain above 1500 m elevation where sub-grid topography dominates and where the validation network has no coverage to constrain skill directly, coastal zones within 20 km of ocean due to unresolved mesoscale processes, and data-sparse regions of northern Myanmar and Laos where underlying climatologies have limited station coverage. Urban heat island effects are not represented due to static land surface characterization. Precipitation bias intensifies post-2000 in some coastal areas; users analyzing long-term trends in these regions should account for potential temporal non-stationarity in bias. The dataset covers 1981–2014 only; extension beyond 2014 requires updated TerraClimate monthly inputs which are available through 2023 but not yet processed into daily format. Grid cells represent approximately 16 km² spatial averages, not point values; direct comparison with station observations should account for spatial scale mismatch.

#### Validation considerations

Independent validation used 77 GHCND stations with known limitations. First, 76 of the 77 stations lie below 500 m elevation (median 35 m, maximum 676 m at Son La, Vietnam), with only one station — Son La — sampling the 500–1000 m band and no station above 1000 m, so the highland belts of the Arakan Yoma, Chin Hills, northern Laos, and the upper Annamites (above 1000 m) are entirely unsampled and skill in these regions is unverified by the present validation rather than confirmed. Second, the network is concentrated in populated lowlands and under-samples coastal, high-elevation, and other complex-terrain sub-regions, so the reported metrics should be interpreted as indicative of MSEA-Daily performance under lowland, data-rich conditions rather than as fully representative of the entire product domain. Third, per-country validation sample sizes are uneven (Thailand n = 47, Vietnam n = 15, Laos n = 7, Myanmar n = 4, Malaysia n = 3, Cambodia n = 1); only Thailand and Vietnam exceed the n ≥ 10 threshold required for robust country-level estimates, while skill values for Laos, Myanmar, Malaysia, and Cambodia are reported with the † indicative-only marker (and the ‡ single-station-fallback marker for Cambodia, n = 1) in Table S1, and should therefore be read as suggestive of likely performance rather than as statistically robust country-level estimates; the country-to-country heterogeneity visible in Fig. 2 reflects this combination of uneven station density, varying terrain complexity, and inherited biases in the CHIRPS and AgERA5 source datasets rather than genuine differences in MSEA-Daily skill alone. Fourth, station records exhibit missing-data rates exceeding 20% for some stations, and point-to-grid representativeness errors — particularly for precipitation, where 4 km grid-cell averages cannot capture sub-grid convective variability — can inflate RMSE relative to true grid-cell skill. Fifth, the high correlation between MSEA-Daily and CHIRPS (precipitation) and AgERA5 (temperature) reported in Source-Data Benchmarking subsection is by design, since these source datasets supply the daily patterns inherited by MSEA-Daily through the disaggregation procedure; users should therefore interpret the internal-consistency checks as quality control rather than as independent validation, and should rely on the GHCND comparisons for genuine accuracy assessment. Taken together, the 77-station network constitutes the primary independent assessment of MSEA-Daily but does not capture performance in data-sparse mountainous areas, remote coastal stretches, or other complex-terrain environments; users with local observations are encouraged to conduct site-specific validation for their region of interest, and the validation metrics reported here should be understood as domain-averaged performance under lowland-network conditions rather than as fully representative of all sub-regions, with local accuracy potentially differing substantially in regions with sparse validation coverage or complex terrain.

TerraClimate, the monthly anchor of MSEA-Daily, has been widely validated and adopted for regional climate research and impact studies. In general, validation studies indicate strong temperature performance but larger precipitation uncertainties in tropical regions and complex terrain^13,43^. The original product was evaluated against approximately 14,000 GHCND stations globally by Abatzoglou *et al*.^13^, who reported monthly Tmax and Tmin RMSE typically below 2 °C and monthly precipitation correlations typically above 0.7 in CRU-rich regions including Southeast Asia. For mainland Southeast Asia specifically, TerraClimate has been adopted as the reference monthly climate baseline in several recent regional impact studies. Vu *et al*.^44,45^ used TerraClimate-derived Palmer Drought Severity Index (PDSI) at ~10 km resolution to quantify drought and flood impacts on croplands across Myanmar, Thailand, Laos, Cambodia, Vietnam, and Malaysia over 1980–2019, providing a continental-scale precedent for treating TerraClimate as the regional climate baseline over our exact study domain. Mohammed & Sasaki^46^ used TerraClimate climate variables in irrigated-crop modelling for Cambodia, and Dau *et al*.^47^ used TerraClimate as the historical observational reference for bias correction of CMIP6 drought projections in the Lancang–Mekong River Basin. TerraClimate is also incorporated as a primary climate dataset in Google Earth Engine and Climate Engine, supporting operational drought and water-balance monitoring across the region. Across these studies the consensus is that TerraClimate accurately reproduces monthly mean temperature and the seasonal cycle of precipitation, with some underestimation of monthly precipitation in narrow mountain valleys and along coastal margins where the ~4-km grid does not fully resolve orographic and sea-breeze convection.

#### Technical considerations

Each 4-km grid cell represents the climatology of TerraClimate, which itself interpolates from coarser source data (CRU Ts4.0 at 0.5°, WorldClim at ~1 km). The effective resolution may be coarser than the nominal 4-km grid spacing in regions where underlying station networks are sparse. Daily variability patterns are constrained by temporal coverage of source datasets (CHIRPS 1981-present, AgERA5 1950-present). The power transformation exponent (α = 1.35) for precipitation was optimized against GHCND stations and may not be optimal for all geographic locations or climate regimes. Temperature daily anomalies preserve AgERA5’s representation of synoptic variability, including any limitations in AgERA5’s ability to capture mesoscale weather features or extreme events. Monthly conservation is guaranteed by construction but does not ensure that individual daily values are equally accurate throughout the month.

## Supplementary information

Supplementary Information

## Data Availability

The MSEA-Daily dataset is publicly archived at Science Data Bank under https://doi.org/10.57760/sciencedb.36048. The dataset comprises four climate variables (precipitation, maximum temperature, minimum temperature, and mean temperature) provided as daily gridded fields at 4-km resolution (1/24°) for mainland Southeast Asia covering the geographic extent of 4–24 °N and 92–110 °E. Temporal coverage spans January 1, 1981 to December 31, 2014, providing 12,418 daily time steps across 408 months. All data are provided in NetCDF

## Code availability

The code for temporal disaggregation of TerraClimate monthly data to daily resolution is publicly available at Science Data Bank (https://doi.org/10.57760/sciencedb.39686). The repository includes generate_daily_terraclimate.py implementing the complete processing pipeline: pattern extraction from CHIRPS (precipitation) and AgERA5(temperature), Numba-optimized disaggregation algorithms, monthly conservation verification, and parallel processing with disk-based caching. The code requires Python 3.8+ with dependencies xarray (≥2022.3.0), numpy (≥1.21.0), numba (≥0.55.0), dask (≥2022.1.0), scipy (≥1.7.0), and netCDF4 (≥1.5.7). Users requiring assistance please contact the corresponding author at teerachai.am@up.ac.th/teerachai4@gmail.com.
