# Supplementary material for: MSEA-Daily: A 4-km daily climate dataset for mainland Southeast Asia (1981–2014)
Source: Sci Data. 2026 Jun 19;13:1386. doi: 10.1038/s41597-026-07653-x (PMC13639013; doi:10.1038/s41597-026-07653-x)
Supplement: Supplementary file 1 [file 41597_2026_7653_MOESM1_ESM.docx]

**Supplementary Methods**

**S1. Spatial Interpolation Details**

**S1.1 Bilinear Interpolation Algorithm**

All source datasets (CHIRPS at 0.05° resolution and ERA5-Land at 0.1° resolution) were remapped to the TerraClimate grid (1/24° ≈ 0.04167° resolution) using bilinear interpolation. For each TerraClimate grid point $(x_{t},y_{t})$, we identify the four surrounding source grid points that form a bounding box:

- $(x_{0},y_{0})$- lower-left corner
- $(x_{1},y_{0})$- lower-right corner
- $(x_{0},y_{1})$- upper-left corner
- $(x_{1},y_{1})$- upper-right corner

The interpolated value at $(x_{t},y_{t})$is computed as:

$$F(x_{t},y_{t})=(1-t_{x})(1-t_{y})F_{00}+t_{x}(1-t_{y})F_{10}+(1-t_{x})t_{y}F_{01}+t_{x}t_{y}F_{11}$$

where:

- $F_{ij}$represents the source grid value at corner $(x_{i},y_{j})$
- $t_{x}=(x_{t}-x_{0})/(x_{1}-x_{0})$is normalized distance in x-direction (0 to 1)
- $t_{y}=(y_{t}-y_{0})/(y_{1}-y_{0})$is normalized distance in y-direction (0 to 1)

This interpolation was applied independently for each daily field to preserve temporal variability patterns rather than interpolating temporal statistics.

**S1.2 Boundary Handling**

**Coastal boundaries:** Grid cells partially over ocean in source datasets but fully over land in TerraClimate grid were handled using nearest-neighbor extrapolation when fewer than 2 valid source cells were available for bilinear interpolation. This affected <1% of domain grid cells, primarily along the Thai-Malay Peninsula and Vietnam coast.

**Domain edges:** For TerraClimate grid cells near domain boundaries (5°N, 22°N, 94°E, 107°E), we extended the source domain by one grid cell in each direction to ensure four valid corners for all interior points. Edge grid cells used linear extrapolation when exterior source data was unavailable.

**Missing data handling:** When source grid cells had missing values (flags in CHIRPS or ERA5-Land), we applied the following priority:

1. If all 4 corners valid → standard bilinear interpolation
2. If 3 corners valid → use triangular interpolation from valid corners
3. If 2 corners valid → linear interpolation between valid corners
4. If <2 corners valid → use spatial mean from 8-nearest neighbors with valid data
5. If no neighbors valid within 50 km → flag as missing in SEA-Daily

Missing data accounted for <0.1% of grid cells during 1981-2014.

**S1.3 Conservation Check for Precipitation**

For precipitation, we verified that spatial interpolation preserved domain-total mass. We computed:

$$M_{\text{source}}=\sum_{i,j} P_{ij}^{\text{source}}\cdot A_{ij}^{\text{source}}$$

$$M_{\text{interp}}=\sum_{i,j} P_{ij}^{\text{TC-grid}}\cdot A_{ij}^{\text{TC}}$$

where $A$ is grid cell area accounting for latitude variation:

$$A(\phi)=R^{2}\Delta\lambda(\sin\phi_{2}-\sin\phi_{1})$$

with $R$= Earth radius, $\Delta\lambda$= longitudinal extent, $\phi_{1},\phi_{2}$= latitude bounds.

Domain-integrated mass conservation error was computed as:

$$\varepsilon_{\text{mass}}=\frac{M_{\text{interp}}-M_{\text{source}}}{M_{\text{source}}}\times100\%$$

Across all daily fields 1981-2014, median $\mid\varepsilon_{\text{mass}}\mid$= 0.08% with 99.5th percentile <0.5%, indicating excellent mass conservation during interpolation.

**S1.4 Interpolation Validation**

To assess interpolation accuracy, we conducted a degradation-restoration test using TerraClimate monthly precipitation:

1. Degraded TerraClimate 4-km data to CHIRPS 5.5-km resolution using area-weighted averaging
2. Restored to 4-km using bilinear interpolation
3. Compared restored field to original TerraClimate

Results showed median absolute difference of 0.12 mm/month (0.8% of mean monthly precipitation) and spatial correlation r = 0.998, confirming minimal information loss from interpolation procedure.

**S2. Precipitation Disaggregation Technical Details**

**S2.1 Power Transformation Rationale**

Monsoon precipitation in Southeast Asia exhibits high intermittency with 50-70% of annual rainfall occurring on 10-15% of days. Simple fractional scaling:

$$f_{\text{raw}}(d)=\frac{P^{\text{CHIRPS}}(d)}{\sum_{d} P^{\text{CHIRPS}}(d)}$$

preserves CHIRPS temporal structure but can underrepresent extreme intensities when monthly totals differ between CHIRPS and TerraClimate. Power transformation with α > 1 concentrates precipitation on fewer, more intense wet days, better capturing monsoon characteristics.

**S2.2 Parameter Optimization Procedure**

**Objective function:** The exponent α was optimized by maximizing:

$$J(\alpha)=0.4\cdot\bar{\text{KGE}}+0.2\cdot(1-\mid\text{relbias}(\text{SDII})\mid)+0.3\cdot(1-\mid\text{relbias}(\text{R95p})\mid)+0.1\cdot(1-\mid\text{relbias}(f_{\text{wet}})\mid)$$

where:

- $\bar{\text{KGE}}$= domain-averaged Kling-Gupta Efficiency against GHCND
- SDII = Simple Daily Intensity Index (mean precipitation on wet days)
- R95p = 95th percentile daily precipitation
- $f_{\text{wet}}$= wet-day frequency (days with P ≥ 0.1 mm/day)
- relbias = $(X_{\text{model}}-X_{\text{obs}})/X_{\text{obs}}$

**Weight rationale:**

- 40% KGE: Overall skill most important
- 30% R95p: Extreme intensity preservation critical for flood/drought applications
- 20% SDII: Mean wet-day intensity important for agricultural water balance
- 10% wet-day frequency: Secondary importance, often well-captured by CHIRPS pattern

**Optimization procedure:**

1. Evaluated candidate values α ∈ {1.0, 1.15, 1.25, 1.35, 1.45, 1.55, 1.70}
2. For each α, generated full 1981-2014 daily dataset
3. Computed J(α) using 68 GHCND precipitation stations
4. Selected α maximizing J(α)

**Results:**

| **α** | $\bar{\text{KGE}}$ | **relbias(SDII)** | **relbias(R95p)** | **relbias(**$f_{\text{wet}}$**)** | **J(α)** |
| --- | --- | --- | --- | --- | --- |
| 1.00 | 0.72 | -0.08 | -0.32 | +0.12 | 0.685 |
| 1.15 | 0.76 | -0.05 | -0.22 | +0.06 | 0.751 |
| 1.25 | 0.78 | -0.03 | -0.16 | +0.03 | 0.782 |
| **1.35** | **0.79** | **-0.02** | **-0.12** | **+0.01** | **0.798** |
| 1.45 | 0.78 | +0.01 | -0.08 | -0.02 | 0.785 |
| 1.55 | 0.76 | +0.04 | -0.05 | -0.06 | 0.757 |
| 1.70 | 0.72 | +0.09 | -0.02 | -0.12 | 0.698 |

Optimal α = 1.35 balances intensity enhancement with frequency preservation, achieving J(α) = 0.798.

**Sensitivity analysis:** Perturbations of ±0.1 around α = 1.35 changed intensity metrics by <5%, KGE by <2%, and wet-day frequency by <3%, indicating stable performance near optimum.

**S2.3 Fragment-Poor Month Handling Algorithm**

**Classification criteria:** A month is fragment-poor if either:

1. CHIRPS monthly total $P_{\text{month}}^{\text{CHIRPS}}\leq\tau_{\text{month}}=1.0$mm, OR
2. Number of wet days (P ≥ 0.1 mm/day) < $k_{\min}=2$

**Treatment:** For fragment-poor months, assign uniform daily fractions before power transformation:

$$f_{\text{raw}}(d)=\frac{1}{\mid m\mid}$$

These uniform fractions then undergo power transformation and scaling as normal. This prevents division-by-zero and ensures reasonable temporal distribution when CHIRPS provides insufficient daily structure.

**Frequency:** Fragment-poor months occurred in 8.2% of month-location combinations, primarily in dry regions (Khorat Plateau, central Myanmar) during November-March dry season.

**S2.4 Zero-Precipitation Fragment Algorithm**

When CHIRPS shows zero precipitation for all days ($P_{\text{month}}^{\text{CHIRPS}}=0$) but TerraClimate has non-zero total ($P_{\text{TC}}(m)>0$), we cannot use CHIRPS temporal structure. This occurred in 2.1% of month-locations, typically in arid areas during dry season.

**Algorithm:**

**Step 1:** Extract climatological wet-day frequency from CHIRPS 1981-2010:

$$f_{\text{clim}}^{\text{wet}}(m,i)=\frac{1}{30}\sum_{y=1981}^{2010} \frac{\text{count}(P^{\text{CHIRPS}}(d)\geq0.1\text{ mm for }d\in m_{y})}{\mid m\mid}$$

where $i$ indexes grid cell spatial location.

**Step 2:** Calculate target wet days:

$$n_{\text{wet}}^{*}=\max(1,\text{round}(f_{\text{clim}}^{\text{wet}}(m,i)\cdot\mid m\mid))$$

Minimum of 1 ensures at least one wet day receives the monthly total.

**Step 3:** Distribute precipitation:

Choose $n_{\mathrm{wet}}^{*}$day indices that are evenly spaced through the month to avoid clustering and to keep the procedure deterministic. Let

$$d_{k}\text{ }=\text{ }1+\lfloor\frac{(k-\frac{1}{2})\text{ }\mid t\mid}{\text{ }n_{\mathrm{wet}}^{*}\text{ }}\rfloor,k=1,\ldots,n_{\mathrm{wet}}^{*},$$

and define the set $W_{t}(i)=\{d_{1},\ldots,d_{n_{\mathrm{wet}}^{*}}\}$. (If any duplicates arise due to month length discretization, increment by $+1$cyclically to ensure uniqueness within $[1,\mid t\mid]$.)

Assign precipitation as

$$P_{\text{final}}(d,i)=\{\begin{matrix} \frac{P^{\text{TC}}(t,i)}{n_{\mathrm{wet}}^{*}(t,i)}, & d\in W_{t}(i), \\ 0, & \text{otherwise.} \end{matrix}$$

**Properties.** By construction,

$$\sum_{d\in t} P_{\text{final}}(d,i)\text{ }\text{ }=\text{ }P^{\text{TC}}(t,i)\text{(mass conservation)},$$

and the realized wet-day fraction is

$$\frac{n_{\mathrm{wet}}^{*}(t,i)}{\mid t\mid}\approx f_{\mathrm{clim}}^{\mathrm{wet}}(m,i)$$

up to a rounding error $\leq\frac{1}{2\mid t\mid}$.

**Validation:** For months where this fallback was triggered, the median absolute deviation between the realized wet-day count and the climatological target was $0.08$days per month (≈6% of the mean monthly wet-day count across affected cases), indicating that the algorithm preserves the CHIRPS-based wet-day climatology while exactly conserving the TerraClimate monthly total.

**S2.5 Monthly Conservation Enforcement**

After disaggregation and any special handling, we apply final conservation correction:

$$\lambda(m,\mathbf{x})=\frac{P_{\text{TC}}(m,\mathbf{x})}{\sum_{d\in m} \hat{P}(d,\mathbf{x})}$$

$$\hat{P}_{\text{final}}(d,\mathbf{x})=\lambda(m,\mathbf{x})\cdot\hat{P}(d,\mathbf{x})$$

**Statistics:** Across all month-locations:

- Median |λ - 1| = 0.0008 (0.08%)
- 95th percentile |λ - 1| = 0.0042 (0.42%)
- 99th percentile |λ - 1| = 0.0089 (0.89%)
- Maximum |λ - 1| = 0.024 (2.4%) at coastal boundaries

This minimal correction demonstrates that power transformation and special case handling preserve conservation well, with small adjustments needed primarily due to numerical precision.

**S3. Validation Metrics and Computation**

**S3.1 Metric Definitions**

All metrics computed between model output **y** and observations **x** with n paired values.

**Bias:**

$$\text{Bias}=\frac{1}{n}\sum_{i=1}^{n} (y_{i}-x_{i})$$

Positive values indicate model overestimation. Units match variable (mm/day for precipitation, °C for temperature).

**Mean Absolute Error (MAE):**

$$\text{MAE}=\frac{1}{n}\sum_{i=1}^{n} \mid y_{i}-x_{i}\mid$$

Measures average magnitude of errors without cancellation. Always positive.

**Root Mean Square Error (RMSE):**

$$\text{RMSE}=\sqrt{\frac{1}{n}\sum_{i=1}^{n} (y_{i}-x_{i})^{2}}$$

Penalizes large errors more heavily than MAE due to squaring. Always positive.

**Pearson Correlation Coefficient (r):**

$$r=\frac{\sum_{i=1}^{n} (x_{i}-\bar{x})(y_{i}-\bar{y})}{\sqrt{\sum_{i=1}^{n} (x_{i}-\bar{x})^{2}}\sqrt{\sum_{i=1}^{n} (y_{i}-\bar{y})^{2}}}$$

Measures linear association. Range: -1 to +1, with 1 indicating perfect positive correlation.

**Nash-Sutcliffe Efficiency (NSE):**

$$\text{NSE}=1-\frac{\sum_{i=1}^{n} (y_{i}-x_{i})^{2}}{\sum_{i=1}^{n} (x_{i}-\bar{x})^{2}}$$

Compares model performance to using observed mean as predictor. Range: -∞ to 1. NSE = 1 is perfect, NSE = 0 means model equals observed mean, NSE < 0 means observed mean is better predictor.

**Kling-Gupta Efficiency (KGE):**

$$\text{KGE}=1-\sqrt{(r-1)^{2}+(\beta-1)^{2}+(\gamma-1)^{2}}$$

where:

- $r$= correlation coefficient
- $\beta=\bar{y}/\bar{x}$= bias ratio
- $\gamma=(\sigma_{y}/\bar{y})/(\sigma_{x}/\bar{x})$= variability ratio

KGE combines correlation, bias, and variability into single metric. Range: -∞ to 1, with 1 being perfect. KGE > 0.5 generally considered good performance.

**S3.2 Deseasonalization Procedure**

For Pearson correlation computation, we removed seasonal cycles to isolate day-to-day variability:

**Step 1:** Compute daily climatology (1981-2014 mean for each calendar day):

$$C(j)=\frac{1}{34}\sum_{y=1981}^{2014} V(j,y)$$

where j = 1 to 365 (366 in leap years), V is variable value.

**Step 2:** Apply 31-day moving average to smooth climatology:

$$\tilde{C}(j)=\frac{1}{31}\sum_{k=j-15}^{j+15} C(k)$$

with circular boundary conditions (day 1 follows day 365).

**Step 3:** Compute anomalies:

$$V^{'}(d)=V(d)-\tilde{C}(j(d))$$

where j(d) is calendar day corresponding to date d.

This removes strong seasonal cycle, allowing correlation to measure fidelity of daily variations rather than seasonal amplitude agreement.

**S3.3 Station Pairing Methodology**

**Spatial pairing:** Each GHCND station was paired with the TerraClimate grid cell containing the station coordinates. For stations within 1 km of grid cell boundaries, we verified pairing using nearest-neighbor check. No stations required manual correction.

**Temporal pairing:** Daily values were paired only when both station observation and SEA-Daily grid value were available (non-missing). Minimum requirement: ≥50% temporal coverage during 1981-2014 (≥6,209 days out of 12,418 total).

**Quality filtering:** Station observations flagged with quality concerns in GHCND were excluded from pairing. Quality flags checked:

- Duplicate dates
- Spatial inconsistency (differs from neighbors by >5 standard deviations)
- Temporal inconsistency (exceeds climatological range)
- Known instrument issues

After quality filtering, median temporal coverage was 78% for precipitation, 85% for maximum temperature, 86% for minimum temperature, and 87% for mean temperature.

**S3.4 Aggregation Across Stations**

**Per-station metrics:** All metrics computed individually for each station using paired daily values.

**Domain averaging:** Station metrics were aggregated using equal weighting to avoid spatial clustering bias:

$$\bar{M}=\frac{1}{n_{\text{stations}}}\sum_{s=1}^{n_{\text{stations}}} M_{s}$$

where $M_{s}$is metric value for station s.

Alternative area-weighted averaging (weighting stations by Thiessen polygon area) produced similar results (differences <3% for all metrics), confirming robustness.

**S3.5 Statistical Significance**

**Correlation significance:** Tested using t-statistic:

$$t=r\sqrt{\frac{n-2}{1-r^{2}}}$$

with degrees of freedom df = n - 2. At 95% confidence (α = 0.05), critical r values:

- n = 1000: r_crit = 0.062
- n = 5000: r_crit = 0.028
- n = 10000: r_crit = 0.020

All reported correlations exceed these thresholds (p < 0.001).

**Bias significance:** Tested using paired t-test whether mean bias differs from zero. All reported biases significant at p < 0.01 except where explicitly noted.

**S3.6 Bootstrap Uncertainty Estimation**

To quantify uncertainty in domain-averaged metrics, we applied station-based bootstrap resampling:

1. Randomly sample n_stations stations with replacement
2. Compute domain-average metric from sample
3. Repeat 10,000 times
4. Extract 2.5th and 97.5th percentiles as 95% confidence interval

Confidence intervals reported in main text derived from this procedure.

**Supplementary Figures**

Figure S1 Per-station Kling-Gupta Efficiency (KGE) of MSEA-Daily against GHCND, 1981–2014. Panels (a)–(d) show each of the 77 in-domain GHCND stations as a coloured marker placed at its lat/lon coordinate, with the colour indicating that station's daily-resolution KGE for the variable shown — (a) precipitation, (b) maximum temperature, (c) minimum temperature, (d) mean temperature. Diverging colour map (red = poor; yellow = moderate; green = good) saturating at KGE = −0.2 and KGE = 1.0. Performance categories (good: KGE > 0.5; moderate: 0 < KGE ≤ 0.5; poor: KGE ≤ 0).

Figure S2. Seasonal probability density functions (PDFs) of MSEA-Daily and GHCND station observations, 1981–2014. Panels (a)–(p) show Gaussian kernel-density estimates for each combination of variable (precipitation, Tmax, Tmin, Tmean; rows) and meteorological season (Cool/Dry DJF, Hot MAM, Rainy JJA, Transition SON; columns), computed by pooling all paired daily (GHCND, MSEA-Daily) values across the 77 in-domain validation stations within each calendar month and then concatenating the three months of each season.

Figure S3 Higher-order distributional statistics comparing MSEA-Daily with GHCND observations: (a) Kolmogorov-Smirnov statistic, (b) skewness difference, (c) excess kurtosis difference, (d) KS test p-values. Dashed line in (d) marks α=0.05 significance threshold.

Figure S4 Independent validation GHCND stations against CHIRPS (for precipitaion), and AgERA5 (for temperature parameters): scatterplots comparing MSEA-Daily with daily station observations for precipitation (PRECIP), maximum temperature (TMAX), minimum temperature (TMIN), and mean temperature (TMEAN) during 1981–2014 across mainland Southeast Asia.

Figure S5 Internal consistency check: MSEA-Daily precipitation compared with CHIRPS source data (1981-2014). (a) daily climatology, (b) monthly climatology, (c) seasonal averages, (d) interannual timeseries, (e) monthly standard deviation, (f) distribution percentiles.

Figure S6. Internal consistency: MSEA-Daily maximum temperature vs AgERA5 (1981-2014) (a) daily climatology, (b) monthly climatology, (c) seasonal averages, (d) interannual timeseries, (e) monthly standard deviation, (f) distribution percentiles.

Figure S7. Internal consistency: MSEA-Daily mean temperature vs AgERA5 (1981-2014) (a) daily climatology, (b) monthly climatology, (c) seasonal averages, (d) interannual timeseries, (e) monthly standard deviation, (f) distribution percentiles.

Figure S8. Internal consistency: MSEA-Daily minimum temperature vs AgERA5 (1981-2014) (a) daily climatology, (b) monthly climatology, (c) seasonal averages, (d) interannual timeseries, (e) monthly standard deviation, (f) distribution percentiles.

Figure S9. Monthly climatology of MSEA-Daily precipitation, 1981–2014. Panels (a)–(l) show the climatological mean daily precipitation (mm day⁻¹) for January through December, computed across all daily values falling in each calendar month over the 34-year record.

Figure S10. Monthly bias of MSEA-Daily precipitation against CHIRPS, 1981–2014. Panels (a)–(l) show MSEA-Daily monthly climatology minus CHIRPS monthly climatology (mm day⁻¹) for January through December.

Figure S11. Monthly climatology of MSEA-Daily maximum temperature, 1981–2014. Panels (a)–(l) show the climatological mean daily Tmax (°C) for January through December.

Figure S12. Monthly bias of MSEA-Daily maximum temperature against AgERA5, 1981–2014. Panels (a)–(l) show MSEA-Daily monthly climatology minus AgERA5 monthly climatology (°C).

Figure S13. Monthly climatology of MSEA-Daily mean temperature, 1981–2014. Panels (a)–(l) show the climatological mean daily Tmean (°C) for January through December.

Figure S14. Monthly bias of MSEA-Daily mean temperature against AgERA5, 1981–2014. Panels (a)–(l) show MSEA-Daily monthly climatology minus AgERA5 monthly climatology (°C).

Figure S15. Monthly climatology of MSEA-Daily minimum temperature, 1981–2014. Panels (a)–(l) show the climatological mean daily Tmin (°C) for January through December.

Figure S16. Monthly bias of MSEA-Daily minimum temperature against AgERA5, 1981–2014. Panels (a)–(l) show MSEA-Daily monthly climatology minus AgERA5 monthly climatology (°C).

Figure S17. Time-latitude evolution: precipitation (1981-2014)(a) CHIRPS, (b) MSEA-Daily, (c) bias (MSEA-Daily minus CHIRPS).

Figure S18. Time-latitude evolution: maximum temperature (1981-2014 (a) AgERA5, (b) SEA-Daily, (c) bias (MSEA-Daily minus AgERA5).

Figure S19. Time-latitude evolution: mean temperature (1981-2014 (a) AgERA5, (b) MSEA-Daily, (c) bias (MSEA-Daily minus AgERA5).

Figure S20. Time-latitude evolution: minimum temperature (1981-2014 (a) AgERA5, (b) MSEA-Daily, (c) bias (MSEA-Daily minus AgERA5

**Figure S21.** Per-pixel correlation between local JJAS anomalies and the JJAS Oceanic Niño Index (ONI), 1981–2014. (a) MSEA-Daily precipitation. (b) CHIRPS precipitation (the upstream daily-pattern source for MSEA-Daily). (c) MSEA-Daily mean temperature. (d) AgERA5 mean temperature (the upstream daily-anomaly source for MSEA-Daily).

**Figure S22.** Lead–lag correlation between MSEA-Daily JJAS regional anomaly and the Niño 3.4 Oceanic Niño Index (ONI), 1981–2014. Each curve shows the Pearson correlation between the MSEA-Daily regional JJAS anomaly and the ONI of every 3-month overlapping season — DJF, JFM, FMA, …, NDJ — both in the same calendar year (suffix "(0)") and in the following year (suffix "(+1)"), giving 24 lags across ±12 months around the JJAS centre. Panels: (a) precipitation, Indochina interior (≥ 12°N); (b) precipitation, Maritime peninsula (< 12°N); (c) mean temperature, Indochina; (d) mean temperature, Maritime peninsula. Open red circles mark seasons with *p* < 0.05; the grey band shows the |*r*| < 0.34 critical region (two-sided *t*-test at *p* = 0.05 for *n* = 34). The dashed vertical line marks the centre of JJAS (lag = 0). The labelled point in each panel is the season of strongest correlation.

**Figure S23.** Standardised JJAS regional anomaly time series for MSEA-Daily, the upstream source dataset, and the GHCND station composite, 1981–2014, with the JJAS ONI on a secondary axis. Panels: (a) precipitation, Indochina (≥ 12°N), comparing MSEA-Daily, CHIRPS and the GHCND 57-station composite; (b) precipitation, Maritime peninsula (< 12°N), comparing the same three datasets over a 20-station composite; (c) mean temperature, Indochina, comparing MSEA-Daily, AgERA5 and the GHCND composite; (d) mean temperature, Maritime peninsula. All time series are JJAS regional means anomalised against the 1981–2010 climatology and standardised to z-scores so the three datasets share a common axis. The JJAS ONI (right-hand axis) is overlaid as semi-transparent bars (red = positive, blue = negative).

**Figure S24.** MSEA-Daily JJAS anomalies regressed against the JJAS Oceanic Niño Index (ONI), 1981–2014. Each panel plots the MSEA-Daily JJAS regional anomaly (computed against the 1981–2010 climatology) on the vertical axis against the JJAS ONI on the horizontal axis, for: (a) precipitation, Indochina (≥ 12°N); (b) precipitation, Maritime peninsula (< 12°N); (c) mean temperature, Indochina; (d) mean temperature, Maritime peninsula. Each circle is one of the 34 JJAS seasons, colour-coded by ENSO class — red = El Niño (JJAS ONI ≥ +0.5 °C), blue = La Niña (JJAS ONI ≤ −0.5 °C), grey = Neutral. The dashed line is the ordinary-least-squares regression of MSEA-Daily anomaly on JJAS ONI; the inset reports Pearson r (asterisk = p < 0.05), regression slope (mm season⁻¹ per ONI unit for precipitation, °C per ONI unit for temperature),

**Supplementary Tables**

Table S1. Stratified independent-validation metrics for MSEA-Daily against GHCND stations (1981–2014), by country, elevation band, and coastal/inland class. † represent n < 10, interpret as indicative.‡ represents single-station daily fallback.

| Stratum | Precipitation | | | | | | Tmax | | | | | | Tmin | | | | | | Tmean | | | | | |
| --- | --- | --- | --- | --- | --- | --- | --- | --- | --- | --- | --- | --- | --- | --- | --- | --- | --- | --- | --- | --- | --- | --- | --- | --- |
|  | r | Bias (mm/day) | RMSE (mm/day) | MAE (mm/day) | NSE | KGE | r | Bias (°C) | RMSE (°C) | MAE (°C) | NSE | KGE | r | Bias (°C) | RMSE (°C) | MAE (°C) | NSE | KGE | r | Bias (°C) | RMSE (°C) | MAE (°C) | NSE | KGE |
| Domain average | 0.61 | -0.96 | 3.25 | 1.51 | 0.3 | 0.36 | 0.96 | -0.4 | 0.66 | 0.51 | 0.88 | 0.92 | 0.94 | -0.28 | 0.63 | 0.52 | 0.86 | 0.94 | 0.97 | -0.02 | 0.42 | 0.29 | 0.93 | 0.95 |
| By country | | | | | | | | | | | | | | | | | | | | | | | | |
| Thailand | 0.93 | -0.69 | 0.93 | 0.78 | 0.72 | 0.83 | 0.78 | -0.51 | 0.71 | 0.54 | 0.13 | 0.78 | 0.96 | -0.46 | 0.62 | 0.54 | 0.82 | 0.94 | 0.92 | 0.01 | 0.37 | 0.23 | 0.85 | 0.92 |
| Malaysia | 0.95† | 0.14† | 0.25† | 0.22† | 0.86† | 0.89† | 1.00† | -0.22† | 0.34† | 0.27† | 0.37† | 0.39† | 1.00† | 0.08† | 0.14† | 0.12† | 0.50† | 0.40† | -1.00† | 0.47† | 0.57† | 0.47† | -1124.50† | -16.23† |
| Laos | 0.66† | -4.53† | 6.45† | 4.53† | -0.28† | 0.13† | 0.99† | 0.06† | 0.55† | 0.46† | 0.82† | 0.60† | 0.66† | -0.07† | 0.87† | 0.75† | 0.21† | 0.64† | 0.94† | -0.53† | 0.62† | 0.56† | 0.48† | 0.80† |
| Myanmar | 0.41† | -2.71† | 10.67† | 8.65† | 0.10† | 0.09† | 0.87† | -0.48† | 0.91† | 0.87† | 0.62† | 0.66† | 0.21† | 0.70† | 0.95† | 0.70† | -1.37† | 0.11† | 0.79† | -0.33† | 0.56† | 0.42† | 0.41† | 0.75† |
| Cambodia | -0.03‡† | -4.08† | 4.08† | 4.08† | —† | -0.20‡† | 0.61‡† | 0.05† | 0.05† | 0.05† | —† | 0.31‡† | -0.06‡† | 0.63† | 0.63† | 0.63† | —† | -0.13‡† | -0.20‡† | -0.33† | 0.33† | 0.33† | —† | -0.21‡† |
| Vietnam | 0.84 | 0.33 | 0.73 | 0.56 | 0.38 | 0.67 | 0.98 | -0.26 | 0.5 | 0.4 | 0.94 | 0.97 | 0.99 | -0.21 | 0.37 | 0.31 | 0.97 | 0.99 | 0.99 | 0.24 | 0.34 | 0.29 | 0.97 | 0.97 |
| By elevation band | | | | | | | | | | | | | | | | | | | | | | | | |
| 0-100 m | 0.62 | -0.78 | 3.34 | 1.56 | 0.35 | 0.41 | 0.97 | -0.37 | 0.58 | 0.5 | 0.9 | 0.89 | 0.87 | -0.17 | 0.61 | 0.5 | 0.74 | 0.78 | 0.97 | 0.01 | 0.35 | 0.26 | 0.93 | 0.94 |
| 100-200 m | 0.78 | -1.38 | 3.63 | 1.66 | 0.26 | 0.22 | 0.98 | -0.36 | 0.47 | 0.38 | 0.89 | 0.94 | 0.71 | -0.33 | 0.58 | 0.49 | 0.25 | 0.68 | 0.93 | -0.06 | 0.37 | 0.26 | 0.85 | 0.83 |
| 200-500 m | -0.76 | -1.09 | 2.1 | 1.12 | -2.94 | -0.79 | 0.92 | -0.53 | 1.07 | 0.78 | 0.79 | 0.89 | 0.8 | -0.58 | 0.8 | 0.66 | 0.24 | 0.73 | 0.91 | -0.08 | 0.69 | 0.49 | 0.82 | 0.87 |
| 500-1000 m | 0.30‡† | 0.64† | 0.64† | 0.64† | —† | 0.27‡† | 0.93‡† | -0.66† | 0.66† | 0.66† | —† | 0.83‡† | 0.96‡† | -0.27† | 0.27† | 0.27† | —† | 0.95‡† | 0.97‡† | 0.22† | 0.22† | 0.22† | —† | 0.93‡† |
| By coast proximity | | | | | | | | | | | | | | | | | | | | | | | | |
| Coastal (< 50 km) | 0.62 | -0.57 | 3.73 | 1.61 | 0.36 | 0.38 | 0.97 | -0.33 | 0.55 | 0.46 | 0.9 | 0.87 | 0.89 | -0.14 | 0.57 | 0.44 | 0.78 | 0.78 | 0.97 | 0.12 | 0.35 | 0.28 | 0.93 | 0.91 |
| Inland (≥ 50 km) | 0.62 | -1.26 | 2.81 | 1.43 | 0.16 | 0.23 | 0.96 | -0.44 | 0.72 | 0.55 | 0.87 | 0.95 | 0.94 | -0.37 | 0.67 | 0.57 | 0.83 | 0.94 | 0.97 | -0.11 | 0.46 | 0.3 | 0.93 | 0.95 |

Table S2. Stratified independent-validation metrics for GHCND stations against CHIRPS (for precipitaion), and AgERA5 (for temperature parameters) (1981–2014), by country, elevation band, and coastal/inland class. † represent n < 10, interpret as indicative.‡ represents single-station daily fallback.

| Stratum | Precipitation | | | | | | Tmax | | | | | | Tmin | | | | | | Tmean | | | | | |
| --- | --- | --- | --- | --- | --- | --- | --- | --- | --- | --- | --- | --- | --- | --- | --- | --- | --- | --- | --- | --- | --- | --- | --- | --- |
|  | r | Bias (mm/day) | RMSE (mm/day) | MAE (mm/day) | NSE | KGE | r | Bias (°C) | RMSE (°C) | MAE (°C) | NSE | KGE | r | Bias (°C) | RMSE (°C) | MAE (°C) | NSE | KGE | r | Bias (°C) | RMSE (°C) | MAE (°C) | NSE | KGE |
| Domain average | 0.65 | -0.87 | 3.12 | 1.35 | 0.36 | 0.38 | 0.91 | -2.05 | 2.20 | 2.05 | -0.36 | 0.89 | 0.92 | 0.16 | 0.79 | 0.58 | 0.79 | 0.84 | 0.94 | -1.16 | 1.30 | 1.16 | 0.34 | 0.90 |
| By country | | | | | | | | | | | | | | | | | | | | | | | | |
| Thailand | 0.96 | -0.55 | 0.73 | 0.57 | 0.83 | 0.86 | 0.81 | -2.23 | 2.32 | 2.23 | -7.64 | 0.58 | 0.93 | 0.13 | 0.64 | 0.49 | 0.81 | 0.84 | 0.91 | -1.14 | 1.23 | 1.14 | -0.69 | 0.80 |
| Malaysia | 0.99† | 0.25† | 0.26† | 0.25† | 0.84† | 0.94† | 0.70† | -2.80† | 2.82† | 2.80† | -57.04† | 0.45† | 0.75† | 0.83† | 0.90† | 0.83† | -3.17† | 0.70† | 0.67† | -0.66† | 0.70† | 0.66† | -8.12† | 0.47† |
| Laos | 0.66† | -4.69† | 6.56† | 4.69† | -0.32† | 0.12† | 0.83† | -1.94† | 2.07† | 1.94† | -1.61† | 0.63† | 0.67† | 0.17† | 0.82† | 0.66† | 0.30† | 0.67† | 0.94† | -2.09† | 2.11† | 2.09† | -5.04† | 0.87† |
| Myanmar | 0.54† | -2.87† | 10.08† | 8.12† | 0.20† | 0.18† | 0.96† | -2.16† | 2.25† | 2.16† | -1.32† | 0.73† | 0.20† | 1.74† | 1.92† | 1.74† | -8.63† | 0.20† | 0.96† | -0.95† | 1.00† | 0.95† | -0.88† | 0.62† |
| Cambodia | -0.05‡† | -3.73‡† | 3.73‡† | 3.73‡† | — | -0.18‡† | 0.49‡† | -0.39‡† | 0.39‡† | 0.39‡† | — | 0.19‡† | -0.04‡† | 0.12‡† | 0.12‡† | 0.12‡† | — | -0.10‡† | -0.20‡† | -1.58‡† | 1.58‡† | 1.58‡† | — | -0.21‡† |
| Vietnam | 0.90 | 0.38 | 0.56 | 0.47 | 0.64 | 0.86 | 0.91 | -1.47 | 1.73 | 1.47 | 0.26 | 0.86 | 0.98 | -0.31 | 0.63 | 0.50 | 0.92 | 0.88 | 0.97 | -0.92 | 1.10 | 0.92 | 0.73 | 0.89 |
| By elevation band | | | | | | | | | | | | | | | | | | | | | | | | |
| 0–100 m | 0.69 | -0.62 | 3.12 | 1.33 | 0.43 | 0.44 | 0.90 | -1.95 | 2.10 | 1.95 | -0.43 | 0.86 | 0.76 | 0.33 | 0.89 | 0.68 | 0.46 | 0.76 | 0.93 | -1.00 | 1.10 | 1.00 | 0.23 | 0.87 |
| 100–200 m | 0.86 | -1.52 | 3.65 | 1.63 | 0.25 | 0.20 | 0.91 | -1.86 | 1.97 | 1.86 | -0.91 | 0.71 | 0.82 | 0.11 | 0.43 | 0.30 | 0.60 | 0.82 | 0.82 | -1.25 | 1.37 | 1.25 | -1.01 | 0.76 |
| 200–500 m | -0.66 | -0.92 | 2.12 | 1.02 | -3.02 | -0.68 | 0.96 | -2.65 | 2.74 | 2.65 | -0.38 | 0.87 | 0.89 | -0.37 | 0.73 | 0.61 | 0.37 | 0.57 | 0.92 | -1.63 | 1.77 | 1.63 | -0.18 | 0.89 |
| 500–1000 m | 0.31‡† | 0.25‡† | 0.25‡† | 0.25‡† | — | 0.30‡† | 0.94‡† | -3.26‡† | 3.26‡† | 3.26‡† | — | 0.82‡† | 0.97‡† | -1.06‡† | 1.06‡† | 1.06‡† | — | 0.88‡† | 0.98‡† | -2.25‡† | 2.25‡† | 2.25‡† | — | 0.89‡† |
| By coast proximity | | | | | | | | | | | | | | | | | | | | | | | | |
| Coastal (< 50 km) | 0.70 | -0.43 | 3.49 | 1.38 | 0.44 | 0.41 | 0.87 | -2.05 | 2.21 | 2.05 | -0.80 | 0.79 | 0.72 | 0.40 | 0.97 | 0.75 | 0.33 | 0.71 | 0.93 | -0.90 | 1.01 | 0.90 | 0.35 | 0.82 |
| Inland (≥ 50 km) | 0.62 | -1.23 | 2.80 | 1.32 | 0.16 | 0.22 | 0.93 | -2.05 | 2.19 | 2.05 | -0.18 | 0.90 | 0.95 | -0.03 | 0.60 | 0.45 | 0.86 | 0.84 | 0.96 | -1.37 | 1.48 | 1.37 | 0.29 | 0.88 |

Table S3. ENSO–MSEA correlation and composite statistics, JJAS 1981–2014. Pearson correlation r and OLS regression slope are between the JJAS regional anomaly and the JJAS Oceanic Niño Index (ONI). El Niño, La Niña and El Niño − La Niña columns are mean JJAS anomalies (relative to the 1981–2010 climatology) over the 9 El Niño (JJAS ONI ≥ +0.5 °C) and 8 La Niña (JJAS ONI ≤ −0.5 °C) seasons. Slope units: mm season⁻¹ / ONI for precipitation and °C / ONI for mean temperature. Anomaly columns: mm season⁻¹ (precipitation), °C (mean temperature). Asterisk on r marks p < 0.05; n = 34 years. MSEA-Daily rows are highlighted in bold for emphasis.

| Variable | Region | Dataset | r | p | Slope | El Niño | La Niña | El Niño − La Niña |
| --- | --- | --- | --- | --- | --- | --- | --- | --- |
| Precipitation | Indochina (≥12°N) | MSEA-Daily | +0.06 | 0.725 | +8.04 | +31.5 | +33.2 | -1.7 |
|  |  | CHIRPS | -0.09 | 0.602 | -10.50 | +12.8 | +24.4 | -11.6 |
|  |  | GHCND | -0.16 | 0.357 | -24.02 | +12.2 | +42.6 | -30.4 |
|  | Maritime (<12°N) | MSEA-Daily | -0.28 | 0.110 | -39.99 | -6.1 | +88.0 | -94.1 |
|  |  | CHIRPS | -0.28 | 0.115 | -24.68 | -11.7 | +38.4 | -50.2 |
|  |  | GHCND | -0.14 | 0.415 | -23.44 | +14.4 | +50.7 | -36.4 |
| Mean temperature | Indochina (≥12°N) | MSEA-Daily | +0.01 | 0.946 | +0.00 | -0.0 | +0.1 | -0.1 |
|  |  | AgERA5 | -0.24 | 0.167 | -0.08 | -0.1 | +0.2 | -0.2 |
|  |  | GHCND | -0.05 | 0.791 | -0.02 | -0.0 | +0.1 | -0.2 |
|  | Maritime (<12°N) | MSEA-Daily | +0.13 | 0.452 | +0.06 | +0.1 | +0.0 | +0.0 |
|  |  | AgERA5 | -0.00 | 0.993 | -0.00 | +0.0 | +0.1 | -0.1 |
|  |  | GHCND | -0.14 | 0.443 | -0.05 | -0.0 | +0.1 | -0.2 |
